# Detection of genomic regions affecting thermotolerance traits in growing pigs during acute and chronic heat stress

**DOI:** 10.1186/s12711-025-00995-x

**Published:** 2025-09-25

**Authors:** Hélène Gilbert, Yann Labrune, Katia Fève, David Renaudeau, Roseline Rosé, Mario Giorgi, Yvon Billon, Jean-Luc Gourdine, Juliette Riquet

**Affiliations:** 1 GenPhySE, Université de Toulouse, INRAE, 31320 Castanet-Tolosan, France; 2 PEGASE, INRAE, Institut Agro, 35590 Saint Gilles, France; 3 ASSET, INRAE, Domaine Duclos Prise d’eau, 97170 Petit-Bourg, France; 4 PTEA, INRAE, Domaine Duclos Prise d’eau, 97170 Petit-Bourg, France; 5 GenESI, INRAE, 17700 Surgères, France

## Abstract

**Background:**

This study aimed to identify genomic regions involved in animal responses to chronic and acute Heat challenges in 1149 pigs tested in three climatic environments (temperate, tropical, and temperate Heated to 30 °C for 3 weeks). Production (growth rate, feed intake and efficiency, backfat thicknesses) and thermoregulation (rectal and cutaneous temperatures) traits were recorded in a backcross between Large White and Créole pigs. Genome-wide association studies were applied to the full population assuming SNP effects to be the same in both environments or to depend on the environment (GxE), and to the population in each environment separately. The genetic models used linkage disequilibrium in all chromosomes (LD) or only in Large White chromosomes (LW), or breed-of-origin of F1 alleles through linkage analyses (LA).

**Results:**

Fifty-two regions distributed on 16 autosomes were detected. Most were identified with the LW or LD analyses, indicating both a large variability of effects in Large White in response to Heat stress, and high variability among the 10 Créole genomes segregating in the design. However, for thermoregulation traits, the majority of QTLs were detected with the LW model, suggesting interesting segregation of susceptibility and resistance alleles within the Large White breed. Ten regions were detected with the GxE model, mainly corresponding to significant effects in the temperate environment and no effect in the tropical situation, except for two regions on chromosome 2, which affected backfat thickness and growth rate, respectively. Twenty-four regions were detected for thermoregulation traits, but none were significant for both rectal and cutaneous temperatures. Of the 13 QTL regions detected for traits recorded during acute stress, four were also detected for similar traits during chronic stress, suggesting some consistency of responses during both stresses, although nine QTL regions were only detected during acute heat stress.

**Conclusions:**

Measuring direct indicators of responses to heat stress, such as thermoregulatory responses, is essential to detect QTL and propose candidate genes involved in these responses. Multiple QTL for thermoregulatory responses segregate in the Large White breed were detected, paving the way for opportunities to select for heat stress resilience in European pig breeds.

**Supplementary Information:**

The online version contains supplementary material available at 10.1186/s12711-025-00995-x.

## Background

Climate change is a major challenge for the future of livestock breeding. Increasing average temperatures and incidence of heat waves [1] are anticipated to have major impacts on the livestock industry [2]. In pigs, these impacts arise from a limited ability of the animals to tolerate heat stress due to low sweating capacities, which rapidly leads to reduced feed intake and associated production outcomes, increased mortality, and welfare issues [3]. Different biological responses to heat stress have been described, depending on the magnitude and duration of the stress, corresponding to chronic and acute heat stresses [4]. A genetic basis of tolerance to heat stress has been reported in most livestock species [5–7]. In contrast to cattle [8] and poultry [9, 10], where multiple studies have searched for genomic regions affecting thermotolerance using genome-wide association studies (GWAS) or for traces of selection, very few studies have examined the genetic determinism underlying those responses in pigs. A first paper reported genomic regions involved in thermotolerance traits in gilts [11], based on crossbred gilts submitted to a control heat stress in experimental conditions. More recently, Tiezzi et al. [12] reported genomic regions involved in the sensitivity of reproductive traits to variations of a temperature-humidity index in Large White and Landrace sows, and Freitas et al. [13] reported regions involved in thermotolerance traits in a large population of Landrace x Large White lactation sows. Both these studies exploited data from commercial conditions under various climatic conditions.

We designed an experiment in the experimental facilities of INRAE to identify genomic regions underlying responses to chronic and acute heat stress in growing pigs and assess whether these responses share genetic mechanisms. The design relied on a backcross population between the Caribbean Creole breed (CR), known to be adapted to tropical conditions but low producing, and the French Large White (LW) breed, selected in temperate conditions for high production and reproduction levels, to exacerbate the variability on the target traits while capturing the within-breed variability. The chronic heat stress was obtained by raising half of the backcross population in a temperate herd (TEMP) and the other half in a tropical herd (TROP). The acute stress was obtained in the TEMP Herd applying a specific 3-weeks heat challenge to the animals. Previous quantitative genetic analyses of these data suggested a genetic basis to responses to chronic heat stress [14, 15] and to acute heat stress [16]. In the present study, GWAS were applied to the production and thermotolerance traits that were recorded during growth, to identify genomic regions affecting quantitative traits (QTL) during chronic heat stress, and during acute heat stress in the TEMP environment only.

## Methods

All measurements and observations on animals were performed in accordance with the law on animal experimentation and ethics at the moment of data collection (CE2012-9 from the Animal Care and Use Committee of Poitou–Charentes and 69-20 12-2 from the Animal Care and Use Committee of French West Indies and Guyana) under the direction of Y. Billon and J. Fleury (INRA-PTEA; authorization number by the French Ministry of Agriculture and Fisheries: 17015 and 971-2011-03 7704, respectively).

### Experimental design and phenotypic traits

This part is briefly described because it has been already detailed in Rosé et al. [15] and in Gourdine et al. [14].

#### Animals

**Fig. 1:**
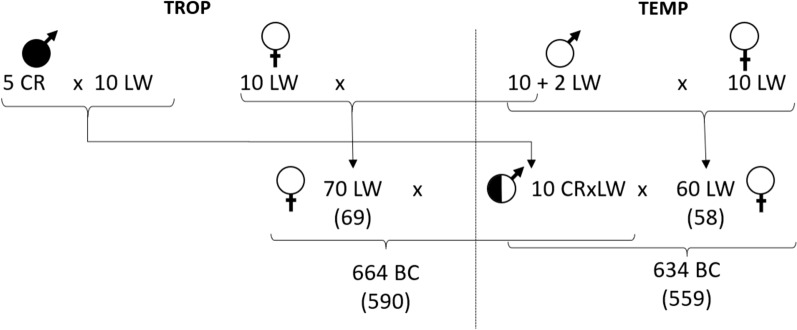
Scheme of the experimental design. Numbers between parentheses indicate the number of backcross (BC) animals genotyped, and the number of F1 dams for these genotyped BC

Data were collected from April 2013 to October 2014 in the experimental facilities of the INRAE experimental unit located in the temperate area (TEMP, INRAE-GenESI, Poitou–Charentes, France; 46° N, 0.45° W, 10.15454/1.5572415481185847E12) and in the semi-opened facilities of the INRAE experimental farm located in the Tropical Platform for Animal Experimentation (TROP, INRAE-PTEA, Guadeloupe, French West Indies; 16° N, 61° W, 10.17180/50N1-KN86). Based on a thermal humidity index (THI), pigs in TROP were exposed to an ambient temperature that exceeded the upper limit of their thermal comfort zone [16]. The backcross design was initiated with 10 F0 purebred LW dams that were inseminated with 5 F0 boars from the CR breed (2 half-sibs, the others being unrelated), which is less productive but more heat tolerant than the LW breed [17], to produce 10 F1 boars (1 pair of half-sibs for 3 of the CR boars, 3 half-sibs for 1 CR boar, and a single progeny of the last CR boar). At the same time, herds of related F1 LW sows were organized in the two farms using artificial insemination on related F0 sows. In order to homogenize the genetic background of the dams, 10 sows in each Herd had from 1 to 3 Litters from the same 10 purebred F0 LW boars across the two herds (except 2 Litters that were from 2 other LW boars in TEMP) (Fig. 1). In total, F1 LW sows were born from 35 Litters, 17 in TEMP and 18 in TROP. Finally, backcross pigs (BC) were produced in each environment, inseminating the related F1 LW sows (60 sows in TEMP and 70 sows in TROP) with semen from the same 10 F1 (CR x LW) boars. The BC were born in 82 Litters in TEMP and in 94 litters in TROP. Each F1 boar produced 7 to 8 Litters in TEMP, and 7 to 11 litters in TROP [15]. In each batch (group of pigs born the same week), 60 (30 females and 30 castrated males) pigs were randomly chosen from eight Litters at 10 weeks of age, balancing sire families and sex ratio. Pigs were penned in groups of 10 pigs of the same sex. They had ad libitum access to a commercial feed that was formulated with the same nutritional characteristics for the two farms (15.7 MJ digestible energy/kg, 170 g of digestible crude protein/kg [15]). Animals had free access to water. The test period started after 1 week of adaptation to the pen.

From 11 to 23 weeks of age, a joint trait recording protocol was applied to pigs in both TEMP and TROP conditions. After 23 weeks of age, a specific 3-week heat challenge was applied to pigs in TEMP to mimic acute heat stress conditions: all pigs were moved to a dedicated room on Friday at the end of week 23, keeping the same group compositions. The next Monday, the room was Heated so the temperature increased by 2 °C every hour, to reach a constant temperature of 30 °C, which was maintained until week 26.

#### Records from 11 to 23 weeks of age in TEMP and TROP

##### Production traits

All animals were weighed at week 11 (BW11) and week 23 (BW23) and average daily gain (ADG) was calculated from week 11 to week 23. Backfat thickness was measured at week 19 (BFT19) and week 23 (BFT23) as the average of ultrasonic measurements (Agroscan, E.C.M, Angoulême, France) on six different positions at 5 cm off the midline, above the point of the elbow, at the last rib (hereafter called P2 site), and at the last lumbar vertebra locations. The average of the two records was computed (BFT1923) and the relative BFT gain from week 19 to week 23 was computed as $${\text{BFTGAINR}} = {\raise0.7ex\hbox{${100{ } \times \left( {{\text{BFT}}23 - {\text{BFT}}19} \right)}$} \!\mathord{\left/ {\vphantom {{100{ } \times \left( {{\text{BFT}}23 - {\text{BFT}}19} \right)} {{\text{BFT}}19}}}\right.\kern-0pt} \!\lower0.7ex\hbox{${{\text{BFT}}19}$}}$$.

Individual feed intake was recorded using single place electronic feeders (ACEMA 128, ACEMO, Pontivy, France). To maximize the number of pigs with feeding measurements, pigs had access to automatic feeders for three periods of 2 weeks during the 12 weeks of test, which alternated with periods of 2 weeks with conventional collective feeders. Hence, average daily feed intake (ADFI) was recorded following two patterns for two groups of pigs: half of the pens had records for period 1 (weeks 11–12, 15–16, and 19–20) and the other half had records for period 2 (weeks 13–14, 17–18, and 21–22). Feed conversion ratio (FCR) was calculated as ADFI divided by ADG. Residual feed intake (RFI) was computed for each animal as the deviation of recorded ADFI from average daily feed intake predicted by a multiple regression of ADFI on ADG between weeks 11 and 23 and of BFT23 as predictors of production requirements of the animal, and the average metabolic body weight during the test as predictor of maintenance requirements, as described in Rosé et al. [15].

##### Thermoregulation traits

Body temperatures were measured at the same time of day (± 1.5 h) for all batches to minimize circadian effects. Cutaneous temperatures were measured on the back at the P2 site at week 19 (CT19) and week 23 (CT23), using a skin surface thermocouple probe (type K, model 88002K-IEC; Omega Engineering, Inc., Stamford, CT) connected to a microprocessor-based handheld thermometer (model HH-21; Omega Engineering, Inc.), and the average of the resulting records was computed (CT1923). Rectal temperatures were recorded in the morning at week 19 (RT19), week 21 (RT21), and week 23 (RT23), using digital thermometers (Microlife Corp., Paris, France), and the average of the three records was computed (RT1923).

#### Records from week 23 to week 26 in TEMP

In addition to the parallel recording protocol in both Herds, animals in TEMP were submitted to a Heat challenge during 3 weeks and additional measurements were recorded at week 24 and week 26. Records at week 24 were obtained on Thursday, i.e., on day 3 after the room reached 30 °C, to capture immediate responses to acute stress, and records on week 26 were taken 2 weeks later. All temperatures were taken on animals in the heated room to Limit the immediate influence of the corridor temperature. All animals were weighed at week 24 (BW24) and week 26 (BW26). Backfat thicknesses at 26 weeks (BFT26) were recorded using the same protocol as described above. Rectal and cutaneous temperatures were measured at 24 and 26 weeks (RT24, RT26, CT24, and CT26 respectively). Then, daily changes in traits were computed between weeks 24 and 23, between weeks 26 and 23, and between weeks 26 and 24 (when the measure at week 24 was available), for instance dBW26-24 $= \left( {{\text{BW}}26{ } - {\text{ BW}}24} \right) /\left( {number\;of\;days\;between\;weeks\;24\;and\;26} \right)$. In addition, daily trait changes relatively to the record at the first measure (at week 23 or 24) were computed, for instance, dBW26-24r = 100 × dBW26-24/BW24. Daily trait changes and daily trait changes relatively to the record at the first measure were similarly computed for cutaneous temperature (dCT24-23, dCT26-23, dCT26-24, dCT24-23r, dCT26-23r, dCT26-24r) and backfat thickness (dBTF26-23 and dBTF26-23r).

Systematic quality control was applied to all recorded and computed phenotypes. Within each environment, records differing by more than three phenotypic SD from the mean (i.e., outliers) were excluded from further analyses [15]. In total 634 BC pigs had records validated in the TEMP environment, and 664 BC pigs in TROP (Fig. 1).

### Samples and genotyping

Blood samples were collected on the F0 parents of the F1 boars, on some of the F0 dams (9 from TEMP and 6 from TROP) of the F1 LW dams, on all F1 boars, on 68 F1 sows with 8 or fewer piglets genotyped, and on 5 F1 sows with 9 piglets. Tail samples were collected at tail docking (2 to 3 days after birth) on all BC pigs. All samples were stored at − 20 °C until DNA extraction. Genomic DNA was purified from these individual biological samples using standard protocols.

Genotypes were obtained at the animal genotyping platform LABOGENA using the Illumina PorcineSNP60 BeadChip platform according to the manufacturer’s protocol. The mapping of the 64,232 SNPs on the pig genome was based on the Sscrofa11.1 assembly of the pig genome (https://www.ensembl.org/Sus_scrofa/Info/Index) [18]. In addition, two known major mutations that may affect the traits of interest were specifically genotyped to be further accounted for in the analyses: the Asp298Asn mutation in the *MC4R* gene [19] and the G3072A mutation of the third intron in the *IGF2* gene [20]. All BC, the F0 CR, the F0 LW dams, the F1 boars and F1 dams with less than 8 piglets (51) were genotyped for these two mutations by allele-specific PCR amplification using the KASPar (Kompetitive Allele-Specific PCR) SNP genotyping system, followed by fluorescence detection on an ABI7900HT Applied Biosystems (end-point fluorescent PCR read). The KASPar assays were carried out in 5 µL reactions following the manufacturer recommendations using KASP v3.0 Master mix 2X (LGC Biosearch Technologies, Middlesex, UK). The distribution of the genotypes for these two mutations is provided in Table 1.

**Table 1:** Distribution of the genotypes for mutations in the *MC4R* and *IGF2* genes

	IGF2			MC4R		
	AA	AG	GG	AA	AG	GG
F0 CR	4	1	0	0	2	3
F0 LW maternal	5	4	6	15	0	0
F0 LW paternal	5	5	0	7	3	0
F1 LW	11	20	20	51	0	0
LWxCR	9	1	0	3	7	0
BC	472	632	45	720	429	0

Quality control was applied to the genotypes. In a first step, a Mendelian error analysis was run using in-house R scripts to identify parent-progeny incompatibilities, which resulted in four BC animals to be removed from the dataset. Then, usual quality control and filtering of the genotypes were applied. Genotypes for SNPs with less than 10% call rate were set to missing for all animals (5720 SNP), then animals with less than 10% genotypes called were set to missing for all SNPs (3 animals), then SNPs with a genotyping call rate lower than 95% were set to missing for all animals (1774 SNPs). In the end, genotypes on 56,738 SNPs on 1262 animals, including the 15 parents of the F1 CR × LW (5 F0 CR, 10 F0 LW dams), 15 F0 dams of the F1 dams, 10 F1 boars, 32 F1 dams from TEMP, and 41 F1 dams from TROP, and 1149 BC (559 in TEMP and 590 in TROP) remained in the dataset. On average, boars had 56 progenies tested and genotyped in TEMP, and 59 in TROP. Finally, SNPS with minor allele frequencies lower than 1% in each environment were discarded.

Imputation was run to obtain the most complete data. Missing genotypes (4.9%) were imputed with the FImpute2.2 software [21] using pedigree information. Only genotypes of SNPs assigned to autosomes were imputed and conserved for Further analyses, leading to a total of 54,231 SNP retained.

Genotypes of BC animals were phased with the LINKPHASE software [22], which uses the pedigree information, to obtain first the parental, then grand-parental, and ultimately the breed of origin of each SNP allele inherited by the BC progeny. The probability of each BC allele to be inherited from the CR paternal grand-sire was stored.

A descriptive plot of the genomic distance between the pigs was obtained using the 43,268 SNPs (call rate and minor allele frequency higher than 5% and a threshold of 10^–10^ for the Hardy–Weinberg equilibrium test), with the multi-dimensional scaling approach (MDS, *cmdscale* function from the STATS package in R), to validate the genomic homogeneity of the full design.

### Statistical analyses

Prior to GWAS, the traits were adjusted for main effects affecting their variability. The effects of sex (2 levels), batch within herd (11 in TEMP and 12 in TROP), and genotypes at the MC4R and IGF2 mutations were tested as fixed effects, as well as the effect of the feed recording period (2 levels) for ADFI and FCR. Various BW were tested as covariates, at the beginning of the test or at the time of measurement. For traits recorded after 23 weeks during the heat challenge in TEMP, similar models were applied, but batch was not nested within herd. Trait abbreviations, resulting number of records, and effects retained in the linear model (P < 0.05) to adjust the traits are summarized in Table 2.

**Table 2:** Trait abbreviations, number of records in each environment^a^, and effects retained in the linear models for adjustment of phenotypes prior to association studies

Abbreviation^b^	N			Covariate	Fixed effects
	ALL	TROP	TEMP		
BW11	1148	589	559		SEX BATCH HERD MC4R
BW23	1147	589	558	BW11	SEX BATCH HERD MC4R IGF2
ADG	1147	589	558	BW11	SEX BATCH HERD MC4R IGF2
BFT19	1148	589	559	BW19	SEX BATCH HERD MC4R IGF2
BFT23	1146	589	557	BW23	SEX BATCH HERD MC4R IGF2
BFT1923	1145	588	557	BW19	SEX BATCH HERD MC4R IGF2
BFTGAINR	1114	569	545		SEX BATCH HERD IGF2
ADFI	1137	586	551	BW11	SEX BATCH HERD IGF2 period
FCR	1136	586	550	BW11	SEX BATCH HERD MC4R period
RFI	1135	586	549		SEX BATCH HERD IGF2
CT19	1148	589	559	BW19	BATCH HERD IGF2
CT23	1147	589	558	BW23	BATCH HERD
CT1923	1148	589	559		BATCH HERD IGF2
RT19	1144	588	556		SEX BATCH HERD MC4R IGF2
RT21	1147	589	558		SEX BATCH HERD MC4R
RT23	1146	588	558		SEX BATCH HERD MC4R IGF2
RT1923	1141	587	554		SEX BATCH HERD MC4R IGF2
BW24	559		559	BW23	SEX BATCH IGF2 MC4R
BW26	559		559	BW23	SEX BATCH IGF2 MC4R
BFT26	559		559	BW23	SEX BATCH IGF2
CT24	559		559	BW23	SEX BATCH IGF2
CT26	559		559	BW23	SEX BATCH
RT24	559		559	BW23	SEX BATCH MC4R
RT26	559		559	BW23	SEX BATCH MC4R
dBW24-23	559		559		SEX BATCH
dBW26-23	559		559	BW23	SEX BATCH
dBW26-24	559		559	BW23	SEX BATCH
dBW24-23r	559		559		SEX BATCH
dBW26-23r	559		559		SEX BATCH
dBW26-24r	559		559		SEX BATCH MC4R
dCT24-23	559		559	BW23	SEX BATCH
dCT26-23	559		559	BW23	SEX BATCH
dCT26-24	559		559	BW23	SEX BATCH
dCT24-23r	559		559	BW23	SEX BATCH
dCT26-23r	559		559	BW23	SEX BATCH
dCT26-24r	559		559	BW23	SEX BATCH
dBFT26-23	558		558	BW23	SEX BATCH
dBFT26-23r	558		558	BW23	SEX BATCH

^a^ TROP, tropical; TEMP, temperate; ALL, TROP + TEMP

^b^ BW, body weight, ADG, average daily gain, BFT, backfat thickness, ADFI, average daily feed intake, FCR, feed conversion ratio, CT = cutaneous temperature, RT = rectal temperature, numbers indicate the week or period of measurement, numbers indicate the week of recording (11, 19, 23, 24, 26), BFT, CT or RT followed by 1923 are the average measurements at 19 and 23 weeks, “d” before BW, CT and BFT indicates the difference between the week records, “r” at the end of the trait abbreviation indicates that the difference is normalized with the first measurement, and $${\text{BFTGAINR}} = {\raise0.7ex\hbox{${100{ } \times \left( {{\text{BFT}}23 - {\text{BFT}}19} \right)}$} \!\mathord{\left/ {\vphantom {{100{ } \times \left( {{\text{BFT}}23 - {\text{BFT}}19} \right)} {{\text{BFT}}19}}}\right.\kern-0pt} \!\lower0.7ex\hbox{${{\text{BFT}}19}$}}$$

#### Genome-wide association studies

##### Models and comparisons

The GWAS were performed using linear mixed models applied to the adjusted phenotypes with the GMMAT software in R [23]. The base model that was applied to each SNP successively was

1 $${\mathbf{y}} = { }{\mathbf{W}}\beta { } + {\mathbf{u}}{ } + { }{{\varvec{\upvarepsilon}}},$$

where $\mathbf{y}$ is the vector of adjusted phenotypes, $\beta$ is the SNP effect, $\mathbf{u}$ is the vector of additive genetic random effects, assumed to follow $N(0,\mathbf{G}{\sigma }_{u}^{2})$, with $\mathbf{G}$ the centered genomic relationship matrix (computed with the GEMMA software, Zhou and Stephens [24]) and ${\sigma }_{u}^{2}$ the additive genetic variance, and **ɛ** the vector of random residual effects, assumed to follow $N(0,\mathbf{I}{\sigma }_{e}^{2})$ with ${\sigma }_{e}^{2}$ the residual variance, and $\mathbf{W}$ is the incidence matrix for the SNP covariate (see next section for description of the SNP effect models).

For traits recorded up to 23 weeks (in both herds), each analysis was first applied on the full population (ALL), comparing the full model to a model under the null hypothesis with $\beta =0$. Then, to determine whether detected genomic regions were subjected to genotype by environment interactions (GxE), two steps were considered: first, the GWAS model was applied to each environment separately (TEMP and TROP). Then, for genomic regions for which a significant (genome-wide threshold) or a suggestive (chromosome-wide threshold) effect was detected in at least one of the environments, the combined data were analyzed with an interaction between the SNP effect and the environment (GxE), using the glmm.gei function in the MAGEE R package, Wang et al. [25].

For traits recorded after 23 weeks during the acute heat challenge in TEMP, each GWAS model was applied to the TEMP animals only, comparing the full model to a model under the null hypothesis $\beta =0$.

Three genetic models for SNP effects were used for the GWAS based on matrix **W**:

- Linkage disequilibrium (LD): LD between SNP and QTL across the parental breeds was tested. Allelic dosage was considered in **W**, with code 0, 1, 2, according to the number of minor alleles of each individual.
- LW segregation (LW): only the effects of inherited LW alleles were tested, considering only progeny that inherited a LW segment from their sire at a tested position (ie LWLW at that position) and genotypes in $\mathbf{W}$ were coded as 0, 1, 2 depending on the number of minor LW alleles the individual carried. On average, half of the BC were considered for this test at each tested position, the other half being LWCR.
- Linkage analysis (LA) based on breed of origin of the F1 allele received by the BC progeny from its sire (corresponding to linkage analyses in the case of a single generation of recombination): genotypes were coded 0 or 1 in $\mathbf{W}$, denoting the number of CR alleles carried at each SNP.

Each type of GWAS model informed on different types segregation of the QTL. The LD GWAS identified SNPs that have consistent LD with QTL across the breeds, while LA GWAS identified QTL alleles that differ in frequency between breeds, and LW GWAS identified QTL segregating within LW.

##### Thresholds

For each SNP and each model, a score test was applied (*glmm.score* in the GMMAT package, R [23]), and the nominal p value was saved. To account for the multiplicity of tests, the number of independent tests applied to each trait was computed, using the methodology described in Nyholt [26]*.* Briefly, for each autosome, a principal component analysis was applied to each SNP genotype correlation matrix ${\mathbf{W}}^{\mathbf{^{\prime}}}\mathbf{W},$ separately for the LD, LA, and LW analyses. The number of principal components corresponding to 99.5% of the total variability on each chromosome was retained and summed over chromosomes. We then used a Bonferroni adjustment to calculate the genome-wide thresholds for a nominal test probability of 5% to identify significant SNPs. Bonferroni was selected due to its conservative control of Type I errors in GWAS, considering that the false discovery rate FDR is unsuitable given the exploratory nature of GWAS based on medium density SNP chips. The resulting genome-wide thresholds for the − log10(p-value) were 5.07, 4.16, and 4.59 for the LD, LA, and LW analyses, respectively. Indeed, with LA analyses, only one generation of segregation was considered, so linkage disequilibrium extended across longer segments than with the LD and LW approaches (see Additional file 1: Figure S1). The same thresholds were applied to the corresponding GxE analyses. Chromosome-wide thresholds were derived using a similar approach and used for discussion of suggestive signals in significant genomic regions (see Additional file 2: Table S1).

##### Grouping significant SNPs into QTL windows and defining QTL regions

For each combination of trait, genetic model (LD, LA, and LW), and population (ALL, TEMP, and TROP, and the corresponding GxE analyses), all significant SNPs were considered to build significant QTL regions. First, for each significant SNP, an interval covering 1 Mb on each side of the SNP (unless the end of the chromosome was reached) was identified and windows were defined as groups of significant SNPs with overlapping intervals (see Additional file 3: Figure S2). In each group, the maxSNP was then identified as the SNP with maximum − log10(p value). Then, if the initial 2 Mb interval of the maxSNP comprised all SNPs in the window, the QTL window was set to this interval. Otherwise, the limits of the intervals of the significant SNPs at the right and left ends of the window were considered to define the QTL window bounds, unless the extremity of the chromosome was reached. These QTL windows defined by trait and analysis were later regrouped into QTL regions when overlapping across analyses or traits, and considered for comparisons across analyses and traits and to search for candidate genes. The estimates $\widehat{\beta }$ and their standard errors (SE) for the maxSNP in each QTL region were obtained with the *glmm.wald* function of the GMMAT package [23].

## Results

### Homogeneity of genomic content in the TEMP and TROP subpopulations

**Fig. 2:**
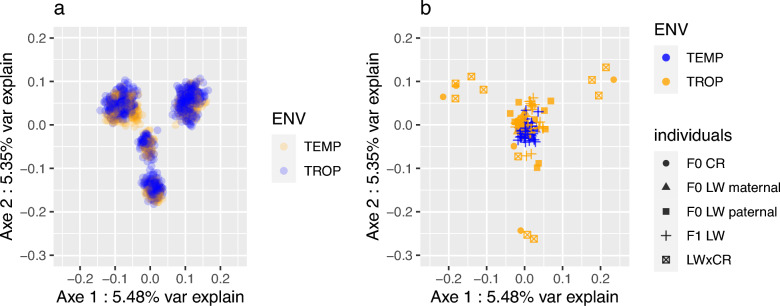
Multidimensional scaling of all individuals in the experimental design. In **a** only backcross individuals are plotted. In **b** only F0 and F1 individuals are plotted

In the MDS applied to all validated genotypes (Fig. 2), the five CR origins of the BC pigs were regrouped in four clusters, as expected from the parental origins. However, the TEMP and TROP subpopulations could not be distinguished within the families, validating the genomic homogeneity in the two herds (Fig. 2a). In addition, the diversity of the population was driven by the CR heterogeneity, with four of the five CR F0 boars projected at the edges of the MDS plan (Fig. 2b), with their F1 progeny nearby, and the LW individuals (F0 and F1) grouped in the middle of the plan. In the plot of the F0 and F1 animals (Fig. 2b), LW parents could not be separated by subpopulation.

### Overview of the detected QTL regions

**Fig. 3:**
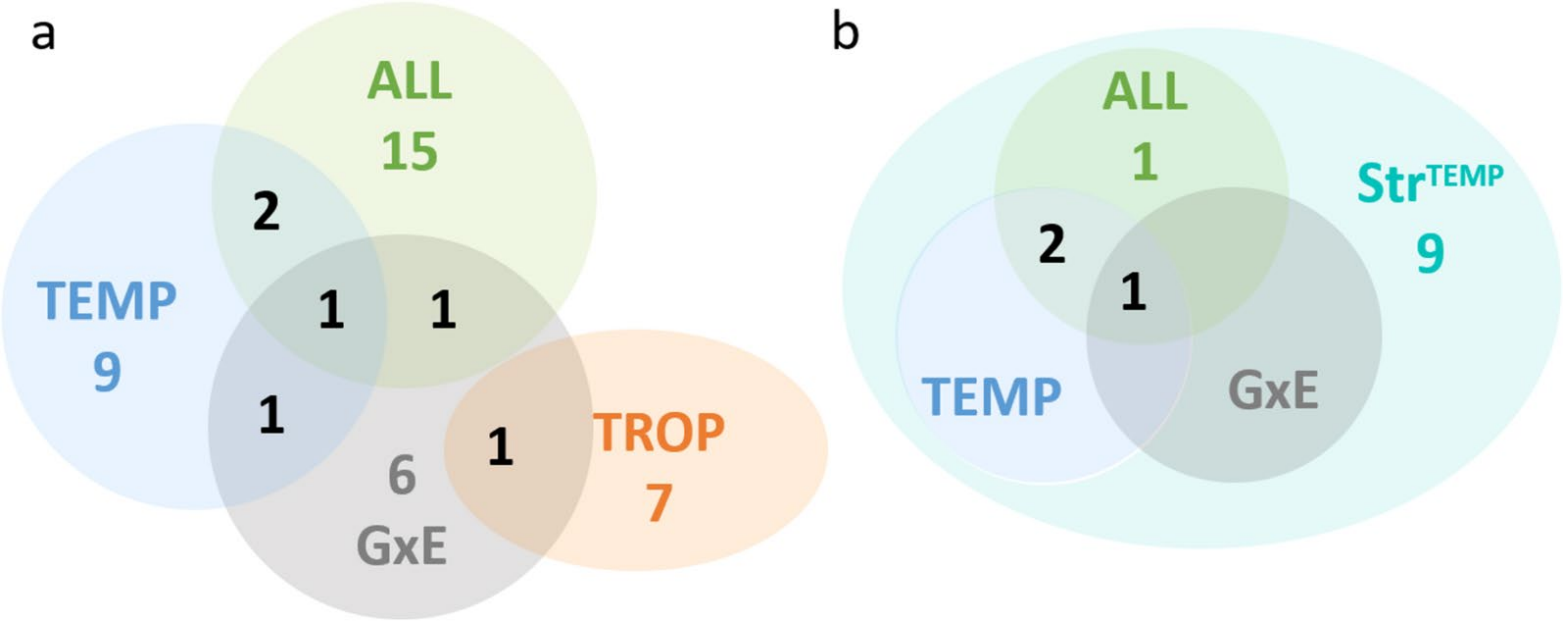
QTL regions detected. **a** Distribution of the 43 QTL regions detected during the chronic stress period in the different analyses. **b** Distribution of the 13 QTL regions detected in the acute stress situation in TEMP in the different analyses. TEMP, QTL regions detected in the temperate environment; TROP, QTL regions detected in the tropical environment; ALL, QTL regions detected in the combined TEMP and TROP data; GxE, QTL regions detected with significantly different effects in TEMP and TROP; Str^TEMP^, QTL regions detected during the acute heat stress in TEMP

**Fig. 4:**
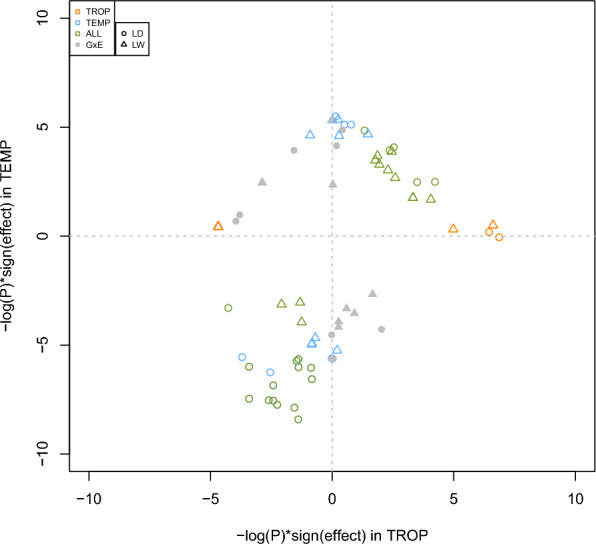
Joint distribution of − log10(P) for QTL identified with the LD and LW analyses in the temperate (TEMP) and tropical (TROP) environments. − log10(P) were multiplied by the sign of the estimated effects, for all significant QTL windows (TROP, TEMP, ALL, GxE). Three QTL regions are missing, as the minor allele frequency of the detected QTL in one environment was too low for analysis in the second environment

First, association studies were carried out to detect QTL for chronic stress (ALL, TROP, TEMP, GxE) for the 17 traits. Ninety-four QTL were identified for 14 traits, 50 detected with the LD model, 37 with the LW model, and 7 with the LA model. Forty-eight were detected with a single significant SNP, the others with 2 to 90 SNPs, defining QTL windows ranging from 2 to 5.5 Mb. Based on all QTL windows detected with the different analyses, 43 QTL regions were defined. Only three of these regions were significant for more than one type of trait (thermoregulation traits, backfat traits, growth and feeding traits) (two regions on chromosomes 2 and 7, SSC2_1 and SSC7_4, for FCR and some BFT traits, and one region, SSC1_1, for BFT and RT), and all other regions were detected for only one type of trait. In most cases, a QTL region was detected with only one model (11 only with the LD model, 23 only with the LW model, and 4 only with the LA model), and few regions were detected by two models (4 regions with both the LD and the LW models, and 1 with the LD and the LA models). All 43 QTL regions were only detected in one environment (Fig. 3a), although two regions were detected in both ALL and TEMP populations. Among the 10 QTL regions detected with the GxE approach, two were significant in TEMP analyses, one in TROP, and two in ALL. Combining the two environments (TEMP and TROP) in the ALL or GxE analyses made it possible to differentiate QTL whose effects had the same sign (ALL) but of different magnitude in the two environments, or opposite sign (Fig. 4).

The analyses for the acute stress (TEMP_Stress) identified 17 QTL windows for 7 traits (BW24, BFT26, dBFT26-23r, RT26, CT26, dCT26-23 and dCT26-23r). These QTLs were exclusively detected with the LD or the LW models (9 with the LD model, 8 with the LW model). The different windows belonged to 13 QTL regions, including 9 that were not detected in the chronic stress analyses. When compared to regions detected for traits recorded during chronic stress, no QTL were detected in the TROP environment, three were detected in TEMP, and four were identified with the ALL dataset, including three regions that were also detected in TEMP, and one that was shared with the GxE analyses (Fig. 3b).

Altogether, 111 QTL windows mapped into the 52 QTL regions were identified. They were distributed on all autosomes but SSC13 and SSC18. The list of the significant QTL regions obtained with the different models (LD, LW or LA) and analyses (ALL, TEMP, TROP, GxE or Stress) are summary in Table 3 for thermoregulation traits, in Tables 4 and 5 for backfat traits and Table 6 for growth and feeding traits. Estimates of QTL effects ranged from 0.24 to 1.85 phenotypic SD (see Additional file 4: Figure S3), equivalent to 2 to 15% of the phenotypic variance (see Additional file 5: Table S2).

### QTL regions detected for thermoregulation traits

**Table 3:** Significant QTL regions for thermoregulation traits, after merging QTL windows for all traits and analyses

SSC	QTL region (start–end in Mb)	Trait^a^	Model-Analysis^ENV^	Nb SNP	Top SNP^b^		
					− l(P)	ID (MAF)	Effect (SE)
1	SSC1_1 (268.4–270.9)	RT1923	LW-ALL	1	4.6	DIAS0000173 (0.47)	0.079 (0.017)
2	SSC2_2 (7.5–9.5)	RT21	LW-TROP	3	5.0	ASGA0008845 (0.03)	0.370 (0.087)
3	SSC3_1 (125.6–127.6)	RT1923	LW-TROP	1	4.9	DRGA0004311 (0.01)	0.396 (0.086)
		RT19	LW-TROP	1	5.2	DRGA0004311 (0.01)	0.543 (0.129)
4	SSC4_1 (7.9–9.9)	RT19	LD-TEMP	1	5.1	MARC0113080 (0.17)	0.132 (0.028)
	SSC4_2 (15.7–17.7)	CT1923	LD-ALL	1	5.1	ALGA0023671 (0.30)	0.128 (0.028)
	SSC4_6 (116.6–118.6)	RT21	LA-TEMP	9	4.3	ASGA0022485 (0.23)	0.132 (0.032)
5	SSC5_2 (23.0–25.0)	RT21	LW-TEMP	1	4.6	DRGA0005536 (0.03)	0.423 (0.098)
	SSC5_7 (87.0–89.0)	RT23	LW-TEMP	4	5.2	ALGA0033410 (0.16)	− 0.197 (0.044)
6	SSC6_4 (81.7–83.7)	RT21	LW-GxE^TEMP^	1	4.6	ASGA0102070 (0.25)	− 0.166 (0.043)
7	SSC7_1 (9.7–11.7)	CT26	LW-Str^TEMP^	1	4.7	MARC0087015 (0.42)	0.335 (0.072)
	SSC7_6 (48.0–50.0)	RT21	LD-ALL	2	5.7	DIAS0004279 (0.14)	0.123 (0.025)
8	SSC8_2 (21.9–23.9)	RT26	LW-Str^TEMP^	1	4.6	DRGA0017702 (0.03)	− 0.427 (0.100)
10	SSC10_1 (12.0–14.0)	RT19	LW-TROP	1	6.6	ALGA0057231 (0.01)	0.642 (0.115)
11	SSC11_1 (37.6–39.6)	CT1923	LW-ALL	1	4.9	ASGA0050675 (0.03)	− 0.409 (0.093)
12	SSC12_1 (23.9–25.9)	CT23	LW-TEMP	1	4.6	H3GA0033927 (0.19)	0.437 (0.105)
	SSC12_2 (25.9–28.3)	RT1923	LD-GxE^TEMP^	8	7.1	ALGA0065768 (0.50)	0.059 (0.015)
14	SSC14_1 (53.4–55.4)	dCT26-23	LW-Str^TEMP^	1	4.9	ALGA0077706 (0.36)	0.023 (0.005)
		dCT26-23r	LW-Str^TEMP^	1	5.0	ALGA0077706 (0.36)	0.072 (0.016)
	SSC14_2 (56.8–60.7)	dCT26-23r	LW-Str^TEMP^	3	5.1	ALGA0077929 (0.22)	− 0.074 (0.016)
		dCT26-23	LW-Str^TEMP^	3	5.0	H3GA0040428 (0.22)	− 0.024 (0.005)
		CT1923	LW-ALL	1	4.6	ALGA0078075 (0.18)	0.214 (0.060)
	SSC14_3 (62.9–64.9)	CT1923	LW-ALL	1	4.7	ALGA0078374 (0.18)	0.217 (0.062)
	SSC14_4 (123.3–125.3)	RT23	LD-ALL	3	5.4	H3GA0042409 (0.25)	0.079 (0.016)
	SSC14_5 (131.8–133.8)	RT21	LW-TEMP	1	5.3	ASGA0067367 (0.02)	0.500 (0.111)
		RT21	LW-GxE^TEMP^	1	4.7	ASGA0067367 (0.06)	0.500 (0.111)
15	SSC15_1 (24.6–26.6)	RT21	LW-TEMP	1	5.4	ASGA0092243 (0.46)	0.172 (0.040)
	SSC15_3 (114.7–116.7)	CT23	LW-ALL	2	4.6	ALGA0086869 (0.19)	0.254 (0.068)
	SSC15_4 (117.5–119.5)	CT1923	LW-ALL	1	5.0	MARC0085247 (0.16)	0.189 (0.056)

LW, QTL regions detected considering allele segregation in the Large White chromosomes only; LD, QTL regions detected considering linkage disequilibrium in all chromosomes; LA, QTL regions detected with linkage association models, contrasting the effects of Large White and Créole alleles; TEMP, QTL regions detected in the temperate environment; TROP, QTL regions detected in the tropical environment; ALL, QTL regions detected in the dataset joining the TEMP and TROP data; GxE^ENV^, QTL regions detected with significant difference of effects between the two environments, with the environment with significant effect indicated (ENV = TEMP or TROP); Str^TEMP^, QTL regions detected during the acute heat stress in TEMP

^a^ see Table 2 for abbreviations for trait names

^b^ For the SNP with max (− log_10_(P-value)) in the QTL region: − l(P) =  − log_10_(P-value); ID = SNP ID; MAF = minor allele frequency in the population of detection; effect (SE) = estimated *β*, with the corresponding standard error into parentheses

**Fig. 5:**
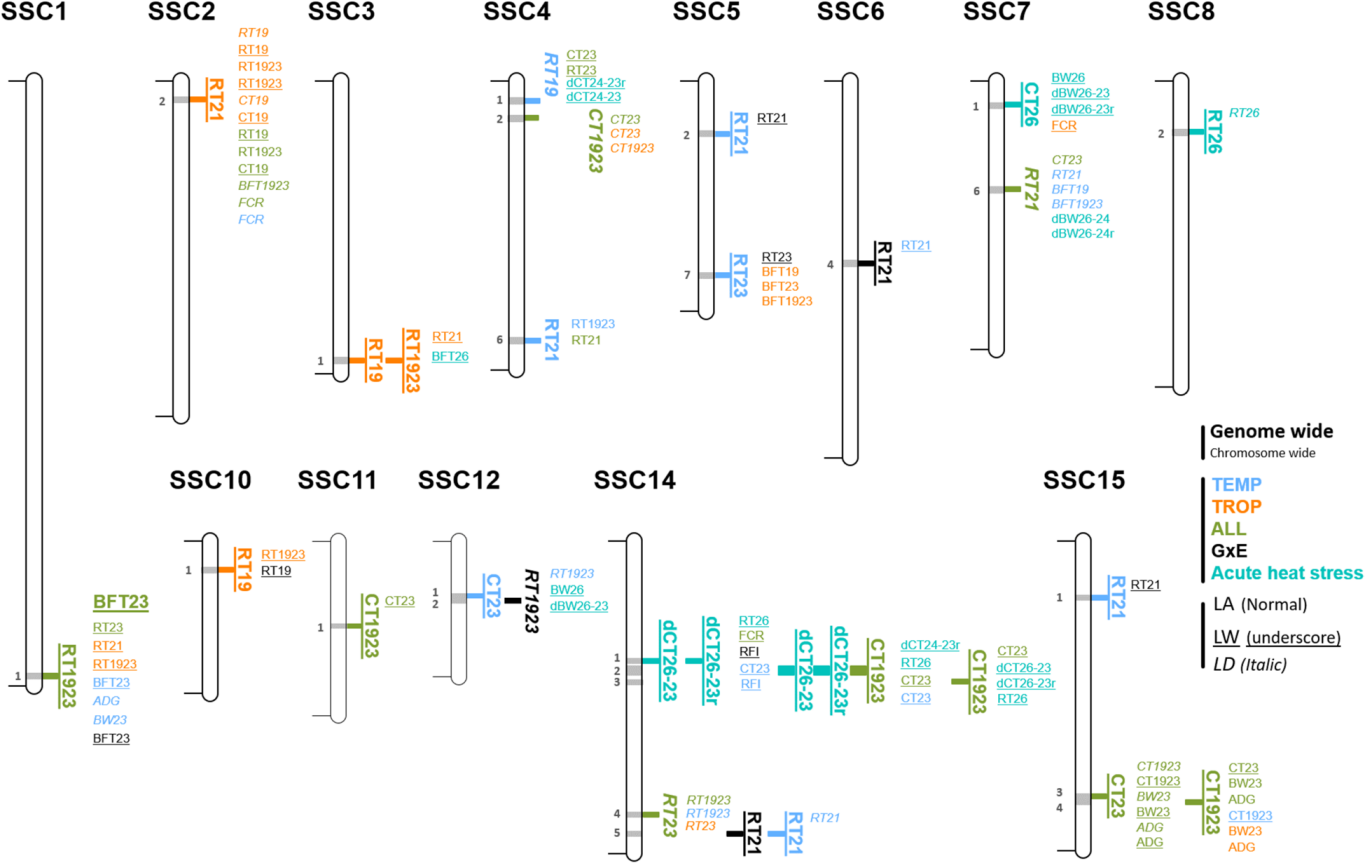
Location of the QTL regions for thermoregulation traits, with the corresponding QTL windows detected with the LD, LW, and LA models in the different analyses (ALL, TEMP, TROP, GxE and acute heat stress). Suggestive QTL windows detected (at chromosome-wide level) in those regions are also shown

Twenty-four QTL regions were identified for thermoregulation traits (Table 3 and Fig. 5). Fifteen regions were associated with rectal temperatures and 9 with skin temperatures. No region showed significant effects for both rectal and skin temperatures. In all 24 regions, suggestive QTL (chromosome-wide level) were identified, that affected different types of traits: 9 suggestive QTL across 5 of the QTL regions detected for thermoregulation also affected BFT traits, and 23 (in 7 QTL regions) also affected growth and feeding traits, and 51 (in all 24 but one) also affected thermoregulation traits. In regions SSC2_2, SSC4_1 and SSC7_6, which exhibited significant effects for rectal temperature, additional suggestive effects for skin temperature were detected. Similarly, in addition to the significant effects detected for skin temperature, suggestive regions for rectal temperature were detected on SSC14 in regions 1, 2 and 3.

For the thermoregulation traits, the majority of significant QTL were detected using the LW model (24 windows in 18 QTL regions) (Fig. 5). Five QTL windows, spread over different regions, were identified using the LD model, and only one significant QTL in the SSC4_6 region was detected using the LA model. This distribution of the outcomes across GWAS models was also found for the suggestive QTL in these regions (47 with the LW model, 23 with the LD model and 13 with the LA model). The QTL regions were significant in only one environment in the single environment analyses. Only the QTL region SSC14_4 showed effects in both environments (TEMP and TROP): significant effects were detected for RT23 with the ALL population, together with suggestive effects for RT1923 in TEMP and RT23 in TROP. For the other regions, the effects were identified in only one environment: 7 regions in TEMP, 3 regions in TROP, and 8 significant regions in the ALL analysis. These last 8 regions had suggestive effects only in one environment (TEMP or TROP).

**Fig. 6:**
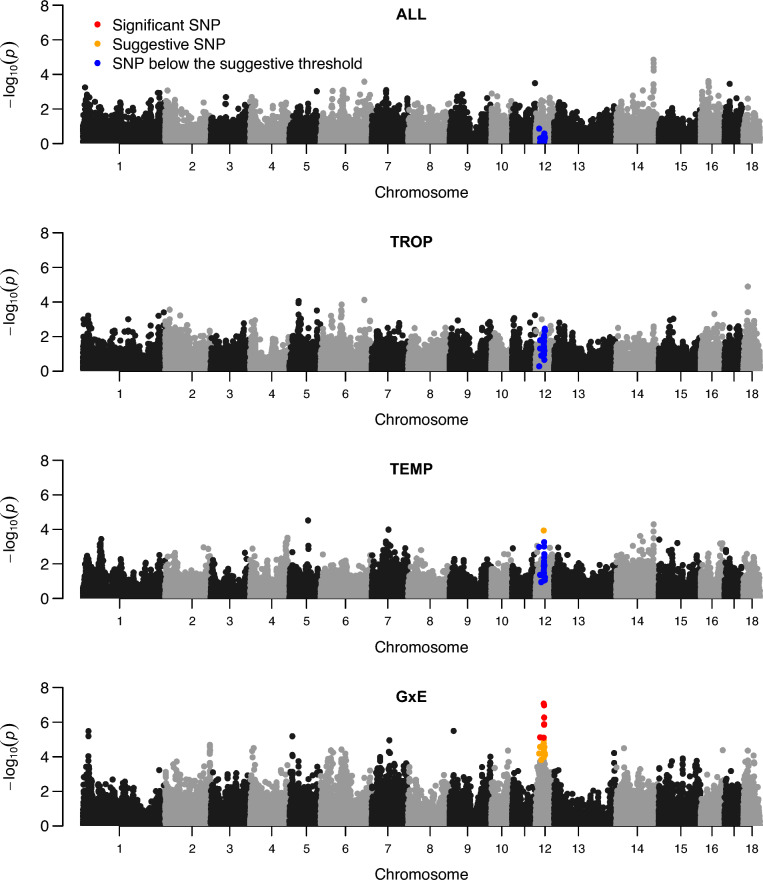
Manhattan plots of the LD analyses to detect genotype by environment interactions (GxE) for average rectal temperature measured at 19 and 23 weeks of age (RT1923), in the combined population (ALL) and in the temperate (TEMP) and tropical (TROP) environments. All suggestive and significant SNPs obtained with the GxE analysis are highlighted in the different Manhattan plots

Three significant QTL windows in SSC6_4, SSC12_2 and SSC14_5 were detected with the GxE model, all for RT measurements (Fig. 5). The 4 suggestive QTL windows that were also identified with these tests also affected RT and were located in other QTL regions (SSC5_1, SSC5_7, SSC10_1, SSC15_1). All these GxE effects were detected using the LW model, excepted in the SSC12_2 region, where the LD model was significant (with an underlying suggestive signal in the TEMP environment (effect = 0.06 °C ± 0.015)) (Fig. 6). For 6 of these regions, QTL effects were significant in TEMP and not in TROP. Only region SSC10_1 showed a significant effect in TROP and not in TEMP.

Four QTL regions were detected for the traits recorded during the acute Heat challenge applied after 23 weeks in the TEMP environment (Table 3). The SSC7_1 and SSC8_1 regions corresponded to specific responses to this acute challenge. The SSC14_1 and SSC14_2 regions also had suggestive effects in the TEMP environment and in the ALL analyses for thermoregulation traits. In addition, suggestive effects for responses to the acute challenge were identified in SSC4_1 and SSC14_3 regions, also detected for RT19 in TEMP and CT1923 with ALL, respectively.

Finally, among the 24 QTL regions detected for thermoregulation traits, 10 presented additional suggestive effects for backfat measures only (SSC3_1 and SSC5_7), for growth and feeding traits only (SSC7_1, SSC12_2, SSC14_1, SSC15_3 and SSC15_4) or for all types of traits (SSC1_1, SSC2_2 and SSC7_6).

### QTL regions detected for backfat traits

**Table 4:** Significant QTL regions for backfat traits, excluding those on SSC7, after merging QTL windows for all traits and analyses

SSC	QTL region (start–end in Mb)	Trait^a^	Model-Analysis^ENV^	Nb SNP	Top SNP^b^		
					− l(P)	ID (MAF)	Effect (SE)
1	SSC1_1 (268.4–270.9)	BFT23	LW-ALL	1	5.0	H3GA0005167 (0.01)	− 2.33 (0.80)
2	SSC2_1 (0.0–5.1)	BFT1923	LD-GxE^TROP^	1	5.8	ASGA0084103 (0.19)	− 0.61 (0.16)
		BFT19	LD-GxE^TROP^	1	6.0	ASGA0084103 (0.19)	− 0.58 (0.15)
		BFT1923	LA-GxE^TROP^	51	4.7	MARC0113696 (0.27)	− 0.44 (0.14)
		BFT19	LA-GxE^TROP^	90	5.7	MARC0113696 (0.27)	− 0.48 (0.13)
4	SSC4_3 (61.4–65.5)	BFT19	LW-GxE^TEMP^	15	6.0	MARC0004593 (0.1)	1.50 (0.45)
		BFT19	LD-GxE^TEMP^	4	5.4	MARC0012316 (0.37)	0.85 (0.21)
	SSC4_4 (83.9–85.9)	BFT23	LD-ALL	1	5.5	H3GA0052602 (0.32)	0.90 (0.19)
	SSC4_5 (106.0–108.0)	BFT23	LD-TEMP	1	5.1	ALGA0027849 (0.44)	0.94 (0.21)
5	SSC5_1 (12.6–14.6)	BFT19	LD-TROP	3	6.9	MARC0091257 (0.25)	0.67 (0.12)
	SSC5_3 (46.0–48.3)	BFT23	LW-ALL	3	5.8	H3GA0016288 (0.14)	− 0.89 (0.25)
	SSC5_4 (53.5–55.5)	BFT23	LW-ALL	1	4.6	ALGA0031988 (0.13)	− 0.92 (0.31)
	SSC5_5 (64.6–66.6)	BFT23	LW-ALL	1	4.7	MARC0023942 (0.34)	0.86 (0.26)
	SSC5_6 (81.0–83.0)	BFT1923	LA-TROP	48	4.7	ALGA0033201 (0.25)	0.64 (0.15)
		BFT23	LA-TROP	49	4.9	ALGA0033201 (0.25)	0.83 (0.19)
6	SSC6_1 (0.3–3.6)	BFT23	LA-ALL	54	4.9	ASGA0105625 (0.26)	0.62 (0.14)
	SSC6_2 (4.7–6.7)	dBFT26-23r	LD- Str^TEMP^	1	5.4	ALGA0034376 (0.23)	− 0.17 (0.04)
9	SSC9_1 (17.6–19.6)	BFT23	LD-GxE^TEMP^	2	5.9	ALGA0051767 (0.16)	− 1.00 (0.24)
	SSC9_2 (32.9–34.9)	BFT1923	LW-GxE^TEMP^	3	6.0	ALGA0052340 (0.29)	− 1.05 (0.35)
17	SSC17_1 (39.9–41.9)	BFT26	LW- Str^TEMP^	1	4.8	ALGA0095080 (0.35)	1.39 (0.35)

LW, QTL regions detected considering allele segregation in the Large White chromosomes only; LD, QTL regions detected considering linkage disequilibrium in all chromosomes; LA, QTL regions detected with linkage association models, contrasting the effects of Large White and Créole alleles; TEMP, QTL regions detected in the temperate environment; TROP, QTL regions detected in the tropical environment; ALL, QTL regions detected in the dataset joining the TEMP and TROP data; GxE^ENV^, QTL regions detected with significant difference of effects between the two environments, with the environment with significant effect indicated (ENV = TEMP or TROP); Str^TEMP^, QTL regions detected during the acute heat stress in TEMP

^a^ see Table 2 for abbreviations for trait names

^b^ For the SNP with max (− log_10_(P-value)) in the QTL region: − l(P) =  − log_10_(P-value); ID, SNP ID; MAF, minor allele frequency in the population of detection; effect (SE), estimated *β*, with the corresponding standard error into parentheses

**Table 5:** Significant QTL regions for backfat traits on SSC7, after merging QTL windows for all traits and analyses

SSC	QTL region (start–end in Mb)	Trait^a^	Model- Analysis^ENV^	Nb SNP	Top SNP^b^		
					− l(P)	ID (MAF)	Effect (SE)
7	SSC7_2 (15.0–17.2)	BFT26	LD-Str^TEMP^	1	5.3	ASGA0031497 (0.07)	− 2.42 (0.50)
	SSC7_3 (19.9–31.1)	BFT1923	LD-TEMP	3	8.4	DIAS0002096 (0.05)	− 2.86 (0.47)
		BFT19	LD-TEMP	2	6.6	DIAS0002096 (0.05)	− 2.34 (0.44)
		BFT23	LD-TEMP	3	7.7	DIAS0002096 (0.05)	− 3.18 (0.54)
		BFT1923	LD-ALL	2	8.0	DIAS0002096 (0.05)	− 1.90 (0.32)
		BFT19	LD-ALL	2	6.2	DIAS0002096 (0.05)	− 1.56 (0.31)
		BFT23	LD-ALL	2	8.3	DIAS0002096 (0.05)	− 2.31 (0.38)
		BFT26	LD-Str^TEMP^	6	8.4	DIAS0002096 (0.05)	− 3.78 (0.61)
		BFT1923	LD-TEMP	1	7.9	ALGA0039628 (0.04)	− 3.07 (0.52)
		BFT19	LD-TEMP	1	6.0	ALGA0039628 (0.04)	− 2.47 (0.49)
		BFT23	LD-TEMP	1	7.5	ALGA0039628 (0.04)	− 3.46 (0.60)
		BFT1923	LD-ALL	1	6.9	ALGA0039628 (0.05)	− 1.91 (0.35)
		BFT19	LD-ALL	1	5.2	ALGA0039628 (0.05)	− 1.51 (0.33)
		BFT23	LD-ALL	1	7.7	ALGA0039628 (0.05)	− 2.41 (0.41)
		BFT1923	LD-TEMP	1	5.6	ASGA0032135 (0.17)	− 1.13 (0.23)
		BFT23	LD-TEMP	1	5.6	ASGA0032135 (0.17)	− 1.34 (0.27)
		BFT1923	LD-GxE^TEMP^	12	8.1	ASGA0032135 (0.16)	− 1.13 (0.23)
		BFT23	LD-GxE^TEMP^	1	7.0	ASGA0032135 (0.16)	− 1.34 (0.27)
		BFT19	LD-GxE^TEMP^	2	5.3	ASGA0032135 (0.16)	− 0.94 (0.22)
		BFT23	LW-GxE^TEMP^	3	5.6	ASGA0032135 (0.09)	− 1.28 (0.52)
		BFT1923	LW-GxE^TEMP^	1	5.3	ASGA0032135 (0.09)	− 0.97 (0.45)
		BFT23	LW-TEMP	1	4.7	ALGA0039856 (0.28)	− 0.85 (0.37)
		BFT26	LD-Str^TEMP^	6	5.5	M1GA0009842 (0.44)	1.3 (0.27)
		BFT23	LD-GxE^TEMP^	1	5.1	ALGA0120268 (0.37)	1.09 (0.24)
		BFT23	LD-ALL	1	5.2	MARC0058766 (0.26)	− 0.92 (0.20)
	SSC7_4 (31.7–35.3)	BFT1923	LD-TEMP	5	7.5	ASGA0032671 (0.05)	− 3.05 (0.53)
		BFT19	LD-TEMP	4	5.6	ASGA0032671 (0.05)	− 2.41 (0.50)
		BFT23	LD-TEMP	7	7.5	ASGA0032671 (0.05)	− 3.49 (0.60)
		BFT1923	LD-ALL	5	7.7	ASGA0032671 (0.05)	− 2.08 (0.36)
		BFT19	LD-ALL	5	5.6	ASGA0032671 (0.05)	− 1.61 (0.34)
		BFT23	LD-ALL	4	8.4	ASGA0032671 (0.05)	− 2.59 (0.42)
		BFT26	LD-Str^TEMP^	7	8.4	ASGA0032671 (0.05)	− 4.23 (0.67)
	SSC7_5 (36.5–44.0)	BFT1923	LD-TEMP	5	6.8	INRA0025164 (0.05)	− 2.89 (0.53)
		BFT19	LD-TEMP	5	6.0	INRA0025164 (0.05)	− 2.51 (0.50)
		BFT23	LD-TEMP	5	6.0	INRA0025164 (0.05)	− 3.12 (0.61)
		BFT1923	LD-ALL	7	7.0	INRA0025164 (0.05)	− 1.98 (0.36)
		BFT19	LD-ALL	6	5.7	INRA0025164 (0.05)	− 1.64 (0.34)
		BFT23	LD-ALL	7	6.9	INRA0025164 (0.05)	− 2.35 (0.43)
		BFT26	LD-Str^TEMP^	7	7.5	ALGA0040849 (0.05)	− 3.99 (0.68)
		BFT1923	LW-TEMP	2	4.7	ASGA0033134 (0.48)	0.60 (0.33)
		BFT1923	LD-TEMP	1	6.3	INRA0025379 (0.05)	− 2.77 (0.53)
		BFT19	LD-TEMP	1	5.7	INRA0025379 (0.05)	− 2.45 (0.5)
		BFT23	LD-TEMP	1	5.6	INRA0025379 (0.05)	− 3.01 (0.61)
		BFT19	LD-ALL	1	5.4	INRA0025379 (0.05)	− 1.60 (0.34)
		BFT26	LD-Str^TEMP^	1	7.2	INRA0025379 (0.05)	− 3.93 (0.69)
	SSC7_7 (56.7–58.7)	BFT26	LD-Str^TEMP^	1	6.4	ASGA0034246 (0.05)	− 3.62 (0.68)

LW, QTL regions detected considering allele segregation in the Large White chromosomes only; LD, QTL regions detected considering linkage disequilibrium in all chromosomes; LA, QTL regions detected with linkage association models, contrasting the effects of Large White and Créole alleles; TEMP, QTL regions detected in the temperate environment; TROP, QTL regions detected in the tropical environment; ALL, QTL regions detected in the dataset joining the TEMP and TROP data; GxE^ENV^, QTL regions detected with significant difference of effects between the two environments, with the environment with significant effect indicated (ENV = TEMP or TROP); Str^TEMP^, QTL regions detected during the acute heat stress in TEMP

^a^ see Table 2 for abbreviations for trait names

^b^ For the SNP with max (− log_10_(P-value)) in the QTL region: − l(P) =  − log_10_(P-value); ID, SNP ID; MAF, minor allele frequency in the population of detection; effect (SE), estimated *β*, with the corresponding standard error into parentheses

**Fig. 7:**
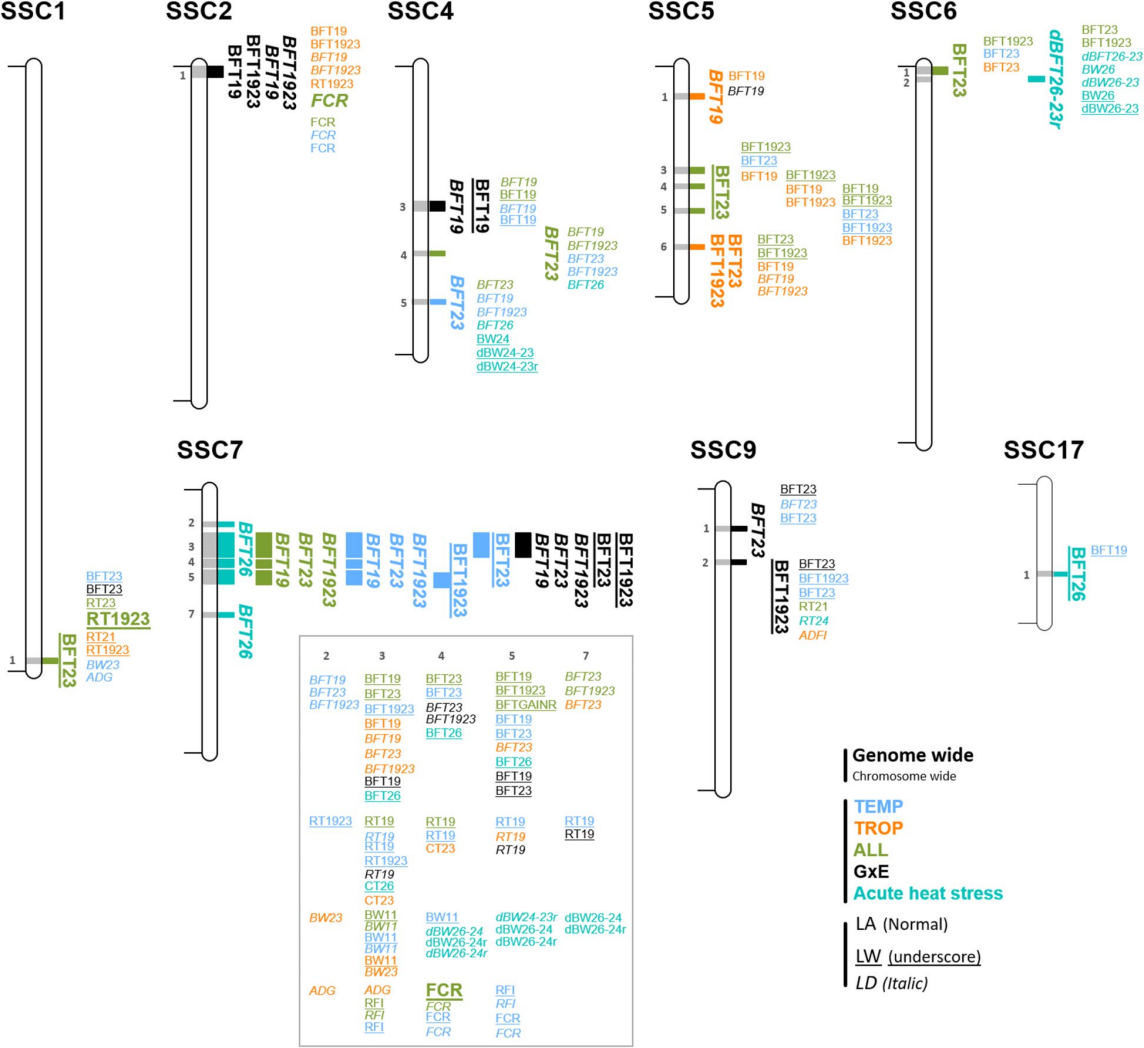
Location of QTL regions for backfat thickness traits, with the corresponding QTL windows detected with the LD, LW, and LA models in the different analyses (ALL, TEMP, TROP, GxE and acute heat stress). Suggestive QTL windows detected (at chromosome-wide level) in those regions are also shown. See Table 2 for trait abbreviations

Backfat traits were the type of trait with the largest number of QTLs detected (Fig. 7). A total of 66 significant QTL windows were identified, grouped into 20 QTL regions (Tables 4 and 5). Of the significant windows, the majority (50) were detected in 10 regions using the LD model, whereas 11 (in 9 regions) were detected using the LW model and 5 (in 3 regions) using the LA model. In three QTL regions, significant effects were also identified for FCR (SSC2_1, SSC7_4) and RT1923 (SSC1_1). In addition, 142 suggestive windows for the different measurements coincided with the 20 BFT QTL regions, including 79 for BFT traits, 41 for growth and feeding traits, and 22 for thermoregulation traits. These suggestive QTL were obtained using the LD (55 QTL in 15 regions), LW (65 QTL in 16 regions), and LA (24 QTL in 13 regions) models. Most of the significant QTL were identified in the ALL data set (15 QTL in 9 regions) or with the GxE analysis (13 QTL in 5 regions). No region was significant in both environments (TEMP and TROP). However, in the SSC7_3 and SSC7_5 regions, significant effects that were detected in TEMP (and ALL) were suggestive QTL in TROP. Besides, the SSC5_3, SSC5_5, and SSC6_1 regions, significant QTL for BFT23 that were detected with ALL also covered suggestive QTL windows detected in both TEMP and TROP. In contrast, 3 QTL regions were specifically detected for responses in TEMP conditions only (SSC4_5) or in TROP conditions only (SSC5_1 and SSC_6_2). Among the 20 QTL regions, seven were detected during the acute heat challenge period. Among these regions, SSC6_2, SSC7_2, SSC7_7, and SSC17_1 correspond to regions that were not significant under chronic stress.

Among all significant QTL windows, two thirds (40 QTLs) mapped to 3 regions on SSC7, SSC7_3, SSC7_4, and SSC7_5 (Table 5). These regions, less than 1.2 Mb apart, were detected for BFT during chronic stress, covered altogether 24.1 Mb, and were mainly detected with the LD model, but also with the LW model. These QTL explained from 4 to 13% (− 3.78 ± 0.61 mm for BFT26) of the phenotypic variance and affected all measures of BFT, i.e. BFT19, BFT23, BFT1923, and BFT26. They were detected either with the full population in the ALL analyses, or with the GxE analyses, with significant effects in TEMP. Multiple suggestive signals were obtained for the same traits and models, including GxE interactions with contrasting effects in the two environments, with either opposite signs (i.e., 0.73 ± 0.38 mm in TEMP vs. − 0.71 ± 0.44 mm in TROP for BFT19) or with the same sign but significantly different in magnitude (i.e., 1.05 ± 0.22 mm in TEMP vs. 0.37 ± 0.16 mm in TROP for BFT23). These regions were also suggestive for almost all other traits measured during the chronic and acute challenges, including cutaneous temperatures (CT23 and CT26), rectal temperatures (RT19 and RT1923), growth traits (BW11, BW23 and ADG, and dBW26-24 and dBW26-24r), FCR, and RFI, including some in TROP and with the LA model.

### QTL regions detected for growth and feeding traits

**Table 6:** Significant QTL regions for growth and feeding traits, after merging QTL windows for all traits and analyses

SSC	QTL region (start–end in Mb)	Trait^a^	Model -Analysis^ENV^	Nb SNP	Top SNP^b^		
					-l(P)	ID (MAF)	Effect (SE)
2	SSC2_1 (0.0–5.1)	FCR	LD-ALL	2	5.3	MARC0053324 (0.13)	0.157 (0.034)
	SSC2_3 (35.1–37.1)	ADFI	LD-TROP	1	6.4	ALGA0013031 (0.45)	125.7 (23.9)
	SSC2_4 (79.6–83.9)	ADG	LW-GxE^TROP^	23	7.0	H3GA0006925 (0.45)	− 11.07 (8.33)
		BW23	LW-GxE^TROP^	26	7.0	H3GA0006925 (0.45)	− 0.917 (0.709)
		ADG	LW-TROP	1	4.7	ASGA0010561 (0.31)	− 24.85 (8.68)
		BW23	LW-TROP	1	4.7	ASGA0010561 (0.31)	− 2.00 (0.74)
6	SSC6_3 (19.5–21.5)	RFI	LD-TEMP	1	5.5	H3GA0055563 (0.24)	134.5 (27.5)
	SSC6_5 (154.8–156.8)	ADG	LW-TEMP	1	4.9	ASGA0089837 (0.09)	− 67.2 (20.2)
		BW23	LW-TEMP	1	5.0	ASGA0089837 (0.09)	− 5.845 (1.748)
	SSC6_6 (162.9–164.9)	BW24	LW-Str^TEMP^	1	5.0	ASGA0099164 (0.39)	− 1.280 (0.286)
7	SSC7_4 (31.7–35.3)	FCR	LW-ALL	1	5.5	ASGA0032827 (0.42)	0.141 (0.036)
8	SSC8_1 (9.9–11.9)	FCR	LA-TROP	29	4.4	DIAS0001942 (0.25)	0.141 (0.034)
15	SSC15_2 (45.8–47.8)	RFI	LW-GxE^TEMP^	1	4.7	DRGA0015125 (0.22)	− 201.9 (48.7)
16	SSC16_1 (30.0–32.0)	BW24	LD-Str^TEMP^	1	5.4	MARC0022283 (0.23)	1.114 (0.230)
	SSC16_2 (58.5–60.5)	ADG	LW-ALL	5	4.7	ALGA0091067 (0.18)	23.85 (8.12)
		BW23	LW-ALL	5	4.8	ALGA0091067 (0.18)	2.06 (0.70)

LW, QTL regions detected considering allele segregation in the Large White chromosomes only; LD, QTL regions detected considering linkage disequilibrium in all chromosomes; LA, QTL regions detected with linkage association models, contrasting the effects of Large White and Créole alleles; TEMP, QTL regions detected in the temperate environment; TROP, QTL regions detected in the tropical environment; ALL, QTL regions detected in the dataset joining the TEMP and TROP data; GxE^ENV^, QTL regions detected with significant difference of effects between the two environments, with the environment with significant effect indicated (ENV = TEMP or TROP); Str^TEMP^, QTL regions detected during the acute heat stress in TEMP

^a^ see Table 2 for abbreviations for trait names

^b^ For the SNP with max(− log_10_(P-value)) in the QTL region: − l(P) =  − log_10_(P-value); ID, SNP ID; MAF, minor allele frequency in the population of detection; effect (SE), estimated *β*, with the corresponding standard error into parentheses

**Fig. 8:**
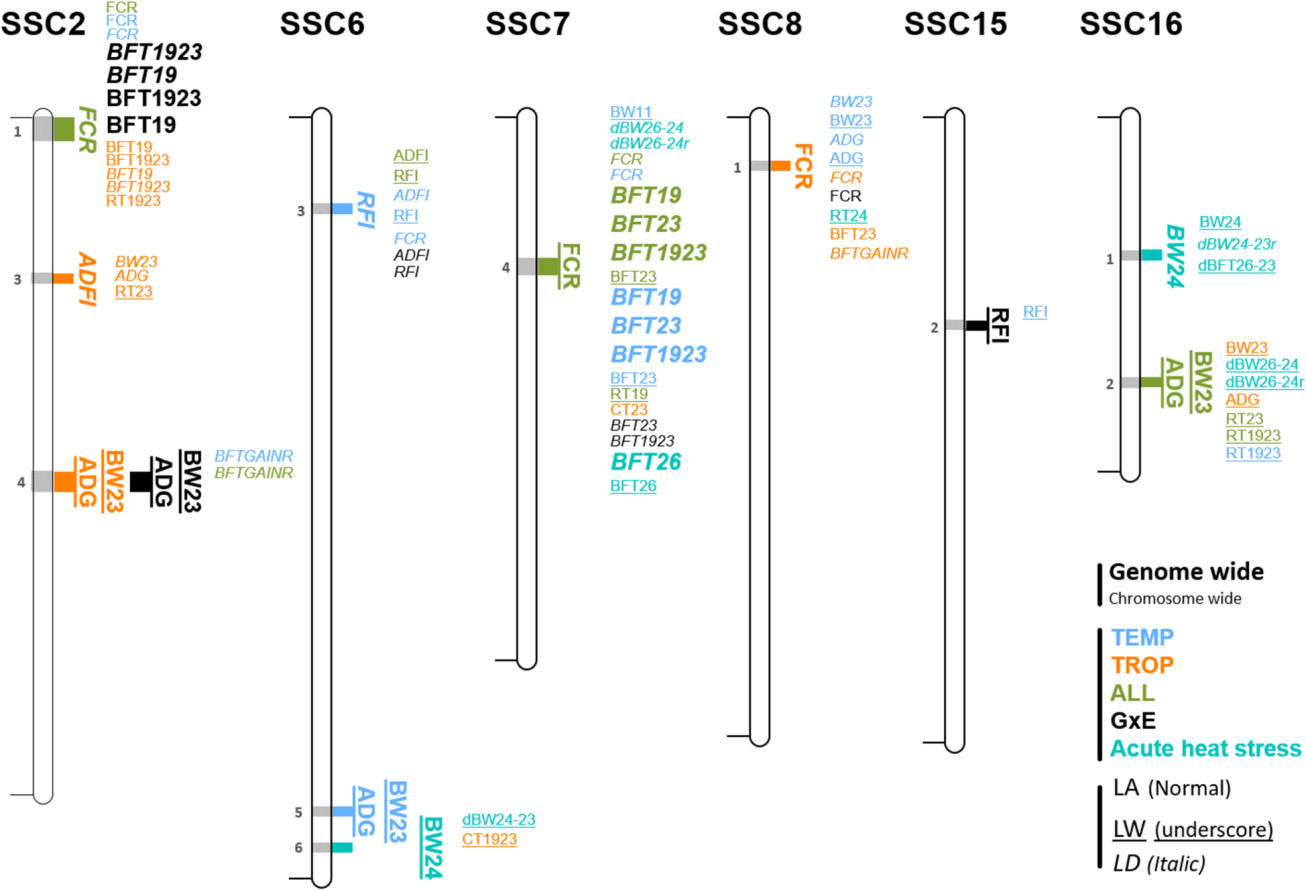
Location of QTL regions for growth and feeding traits, with the corresponding QTL windows detected with the LD, LW, and LA models in the different analyses (ALL, TEMP, TROP, GxE and acute heat stress). Suggestive QTL windows detected (at chromosome-wide level) in those regions are also shown. See Table 2 for trait abbreviations

Five QTL regions were detected for growth traits (ADG and body weights) and 6 for feeding traits (Table 6), which comprised 16 significant windows detected for these traits, and 11 for other types of traits (Fig. 8). Most QTL windows were identified with the LW model (11), while 4 were detected with LD and 1 with LA. Among these regions, SSC2_1 and SSC7_4, which were detected for FCR in the ALL data set, also affected BFT traits. Their effects on FCR accounted for 3 to 5% of the variance of the trait. Three regions (SSC2_3, SSC2_4 and SSC8_1) were detected in TROP: in SSC2_3, suggestive peaks were only obtained in TROP for other traits, while in SSC2_4, GxE interactions were significant, associated with effects on ADG and BW23 in TROP only. This last region also contained suggestive windows for BFTGAINR in TEMP (and ALL). Finally, the SSC8_1 region, detected for FCR in TROP, covered a suggestive GxE signal for this trait, plus additional suggestive effects for growth traits in TEMP. In contrast, two regions on SSC6 were only detected in TEMP: SSC6_3, for RFI, which comprised suggestive peaks for FCR and ADFI, and SSC6_5 with significant effects on ADG and BW23. In addition to SSC2_4, SSC15_2 was identified for significant GxE interactions affecting RFI, with an estimated effect in TEMP of −191 ± 41 g/d. Finally, two QTL regions were detected for growth traits during the acute heat stress: SCC6_6 and SSC16_1. They were not detected in other analyses.

## Discussion

### Design, advantages and limits

#### A homogeneous population built with success

The experimental population was organized to establish two genetically homogeneous populations in the TROP and TEMP environments. Genomic distances between individuals from the two subpopulations (Fig. 2) validated the approach, as groups corresponded to sire families only, and climatic environments could not be distinguished based on genomic information. Thus, differences observed in the GWAS are Linked to differences in the genetic basis of traits between the two environments and not to genetic differentiation between the two subpopulations, which could effectively lead to the identification of different expressions of QTL alleles depending on the environment. Indeed, 10 QTL regions were detected with the GxE approach, which was the least powerful model used in our analyses.

#### Limited specific effects of the CR chromosomes, but large genetic diversity in LW for traits involved in response to heat stress

Very few regions were detected with the LA model, which, based on the hypothesis of differences in QTL allele frequencies between the CR and LW breeds, was the most powerful model in our analyses. In addition, a large genomic distance was found between in the F0 CR genomes in our design (Fig. 2b). These two outcomes suggest that different QTL alleles segregate in CR, so the LA model, which considers that different QTL alleles segregate in CR vs in LW chromosomes, had low power. In practice, the F0 CR boars were selected from a sampling of 38 pigs in the early 2010, to represent the 5 groups identified in the diversity of this small population [27]. This strategy probably enhanced the genetic diversity of the few CR chromosomes that segregated in our design. However, the LD model managed to identify some QTL regions, suggesting similar QTL effects and linkage disequilibrium in the CR and LW chromosomes in some cases. In the few cases where QTL regions were detected with the LA model, the CR alleles increased the values of the trait compared to LW alleles (RT21 on SSC4_6 in TEMP, BFT1923 and BFT23 on SSC5_6 in TROP, BFT23 on SSC6_1 in ALL, and FCR on SSC8_1 in TROP). These directions were consistent with the breed differences previously reported between CR and LW (higher BFT and lower feed efficiency in CR), except for rectal temperatures, as CR and LW sows bred in thermoneutral conditions (under 24.5 °C) did not have different RT [28]. When GxE was significant for the LA analyses (SSC2_1 for BFT traits), the CR alleles decreases trait values compared to LW alleles in TROP (about − 0.45 ± 0.14 mm), but the opposite was true in TEMP (about + 0.50 ± 0.19 mm). The same region was suggestive with the LA analyses for FCR in TEMP, suggesting that, compared to the LW alleles, CR alleles increase FCR in TEMP.

In addition, an important number of the QTL regions were detected with the LW model (45 out of 111), suggesting interesting variability of alleles affecting traits of interest within the LW breed. For thermoregulation traits, 80% of the QTLs were detected using the LW model. Unlike the other two types of trait, we can hypothesise that, despite past selection on production traits, some interesting genetic variability still remains in this breed, unlike the CR breed, which is highly adapted to tropical conditions [29]. This high variability within the LW population also contributed to the relatively lower number of regions detected with LA and LD models for these thermoregulation traits. This confirms reports from the literature in other species or stages, where some genetic variability within mainstream commercial livestock breeds or lines was reported for thermoregulation traits, as in Holstein cows [30], brown laying hens [31], and in LW sows [32]. However, to date, limited reports were available in commercial purebred pigs, as studies focused on crossbred animals at different life stages (gilts: [11], growing pigs: [14, 33]).

### General outcomes of the association studies

#### Detection power

The design was set up after a power analysis by simulation, focusing mainly on the contrast of effects between the CR and LW alleles. From this preliminary study, with QTL alleles having a frequency of 0.25, it was quite unlikely to detect QTL effects lower than 0.30 phenotypic SD. Indeed, these represented only 9% of the detected windows (i.e., 7 QTL windows out of the 77 corresponding to QTL with no GxE).

#### QTL detected for traits indicative of response to heat stress with some GxE interactions

In the study to estimate the genetic components of the chronic stress traits in our design, Gourdine et al. [14] reported significant genetic variances for all growth, feeding, and backfat traits, and for rectal temperatures. In addition, based on genetic correlations differing from 1 between environments, significant GxE was identified for all traits but FCR and ADG [14]. In the present study, QTL in the ALL, TEMP and TROP environments were detected for all types of traits recorded during the chronic heat challenge, but with a majority of QTL windows detected for backfat thickness (14.3 per trait), while fewer QTL were detected for traits that are expected to be more affected by Heat stress, i.e. temperature and feeding traits: 3.25, 2.33, 1.7 and 2.0 windows for RT, CT feeding, and growth traits, respectively. It is worth noting that, although estimates of genetic variances were low for cutaneous temperatures in these data [14], some QTL were identified for these traits in our analyses. Similarly, although the GxE analyses had a smaller power of detection, 20 QTL windows were detected with this model, mainly for BFT traits (14 windows, for 3 traits out of 4), but also 3 for rectal temperatures (for 2 traits out of three), 2 for growth rate (for 2 traits out of 3), and one for RFI (out of three feeding traits). It is also noticeable that only half of the QTL for thermoregulation traits were also significant for production traits, although these traits were previously estimated to have significant genetic correlations in our design [14]. This suggests that specific biological mechanisms control the genetic variability of responses to heat and that dedicated measures directly indicative of thermoregulation responses are needed to study them.

#### Limited specific effects in TROP compared to TEMP during the chronic stress

Despite significant heritability estimates in the two environments for most traits [14], and similar detection power, the number of QTL windows detected in TEMP (30) during the chronic stress was three times greater compared to the number detected in TROP (11). This result has to be compared to the 33 QTL windows detected with the ALL population. Similarly, the regions detected to have GxE were essentially explained by effects in the TEMP environment (8 of the QTL regions), while only two regions on SSC2 (SSC2_1 and SSC2_4) arose from a peak in the TROP environment. This difference could be explained by the numerically smaller genetic variances in TROP compared to TEMP for most traits [14]. In addition, changes in environmental conditions could have impacted pig responses in TROP, as the semi-open building exposed the animals directly to the contrasted “humid” versus “dry” seasons (see Fig. 2 in [15]). Note that presence of GxE as a result of tropical seasons would also erode the power of our design to identify specific effects in TROP.

#### Consistency in the genetic determinism of responses to chronic and acute heat stress

Of the 13 QTL regions detected for traits recorded during the acute stress, 4 were also detected during chronic stress, for similar traits. For instance, the SSC14_2 region was detected for cutaneous temperatures during both types of stress. This region was located less than 2.2 Mb from SSC14_1 (upstream) and SSC14_3 (downstream), which were associated with CT under acute stress and CT under chronic stress, respectively. This suggests a unique region on SSC14 spanning from 53.3 to 64.9 Mb that segregates in the LW population and is involved in heat loss regulation. If confirmed, that QTL could specifically affect the redistribution of blood flow to the periphery of the animal's body. In our population, this region accounted for 5 to 10% of the phenotypic variance of the trait, corresponding to a difference of about 0.40 °C in CT between individuals that carried different alleles.

The three other regions detected under both types of heat stress were SSC7_3, SSC7_4, and SSC7_5, for backfat traits. These regions were also close to each other, distributed from 19.9 to 44.0 Mb and separated by less than 1.2 Mb. They were located close to SCC7_1 and SSC7_2, which were associated with CT and BFT, respectively, during acute stress. These SSC7_3, SSC7_4, and SSC7_5 regions were associated with the larger QTL effect estimates, from 0.24 to 1.33 phenotypic SD of the traits. Most of these QTL were identified with the LD model. When the LW analyses were significant, QTL were also detected with the GxE approach, with estimated effects in the TEMP environment only. In the SSC7 QTLs, some associations were also detected with feed efficiency traits (FCR and RFI), with both the LD and LW models. This chromosome has been reported multiple times as affecting, among others, fatness, growth, and feeding traits in pig QTL studies (2513 entries in the pigQTL database, access 6/2/2024, Hu et al. [34]). Between 19.7 and 33.6 Mb, Fu et al. [35] reported significant associations with skin thickness in an advanced Large White × Tongcheng intercross, with alleles that decreased backfat associated with increased skin thickness. In addition, He et al. [36] in a three way cross Duroc × Landrace × Yorkshire, and Zhang et al. [37] in an F2 Yorkshire x Meishan cross, detected QTL for skin weight around 25.2 and 39.6 Mb, respectively. The CR pigs have thicker dermis than LW pigs, which is associated with differences in other skin characteristics [17] and could contribute to the differences in responses to heat stress detected with the LD model. In addition, this region harbors the swine leukocyte antigen class I and II genes. The massive detection of QTL in this area in our design could be linked with responses to heat stress having common features with immune responses, as reported in Mayorga et al. [38].

The other regions identified to be associated with response to acute heat challenge were smaller (one significant SNP), and two of them did not show suggestive QTL for traits recorded during the chronic stress (SSC8_2 and SSC16_1). This could indicate that these regions were specific to the biological responses to an acute heat stress.

### Candidate genes in regions of interest for responses to heat stress

Metabolic and behavioural changes are evident when pigs are exposed to heat stress [28]. However, thermoregulation is a complex physiological process involving multiple and coordinated physiological mechanisms in different tissues. The list of candidate genes in each QTL region is therefore potentially long. Differential expression analysis studies can be used to target genes with a profile of over- or under-activation under heat stress conditions. Among the published studies, the work of Huau and collaborators [39] has the advantage of being based on the analysis of Large White and Creole animals of same genetic origin and raised in the same facilities as the animals of our study. Among their List of 2008 differentially expressed genes in adrenal glands, blood, Liver, muscle, sub-cutaneous adipose, and thyroid tissues, 179 were located in one of our QTL regions (see Additional file 6: Table S3). Some of these genes are of particular interest, as their functions relate to major metabolic processes identified in responses to acute or chronic heat stress, such as cell division arrest, cell death via apoptosis, necrosis, or autophagy [40, 41].

A first set of genes is related to checkpoints pathways at the G1/S and G2/M transitions of the eukaryotic cell cycle, which respond to damage due to stress by stopping cell cycles, providing time for repair, and by inducing transcription of genes that facilitate repair [42]. These different steps are controlled by CDKs, a large family of heterodimeric serine/threonine protein kinases [43]. Among this family, *CDK1* and *CDK10* are localized in our SSC14_3 and SSC6_1 QTL regions, respectively. In addition to the attention paid to this gene family in numerous cancer research projects [44, 45], research has recently focused on understanding their roles in the adaptive metabolic response to cellular stresses, for instance via the endolysosomal pathway, but also in the functions and dynamics of mitochondria [46]. Mitochondria are indeed highly dynamic organelles and nutritional stress shifts the fission–fusion balance and alters mitochondrial morphology and bioenergetic states. Many mitochondrial genetic disorders are caused by mutations in genes in the nuclear DNA (nDNA) and mitochondrial DNA (mtDNA) that encode structural mitochondrial proteins or proteins involved in mitochondrial function [47]. Among the essential component of mitochondrial respiration, Coenzyme Q (CoQ) has antioxidant properties and helps to mitigate oxidative stress by neutralizing ROS [48]. Among genes that encode CoQ biosynthesis proteins, *CoQ4*, located in the SSC1_1 region of our study, stabilizes complex Q and is needed for efficient CoQ biosynthesis. CoQ4 mutations cause a broad spectrum of mitochondrial disorders, such as bradycardia, respiratory insufficiency, and heart failure associated with CoQ10 deficiency [49]. An impaired CoQ4 function could reduce the effectiveness of this protective mechanism, exacerbating oxidative damage during heat stress.

*HSP90AB1*, which maps to the SSC7_5 QTL region in our study, is also an important candidate gene. In 2001, Nakai and Ishikawa reported that HSFs (heat shock factors) play a role in enhancing cellular survival and adaptation to stress conditions. By inducing the expression of protective proteins, they help cells recover from stress and maintain homeostasis [50]. Nowadays, it is known that HSP90 is engaged in various cellular key functions, including signal transduction, protein folding, protein degradation, cell proliferation, differentiation, and apoptosis [51]. In addition, the HSP70 and HSP90 systems physically and functionally interact to ensure cellular proteostasis. In complement, the *STIP1* (stress-induced-phosphoprotein 1) gene, located in the SSC2_2 region of our study, was described as a eukaryote-specific co-chaperone, which facilitates substrate (“client”) transfer from Hsp70 to Hsp90. It is a good candidate gene for this QTL region.

Heat stress also heavily impacts behaviour. The *PMCH* (pro-melanin concentrating hormone) gene, involved in regulating circadian rhythms and located in the SSC5_6 region, can also be retained as a candidate gene. The product of this gene is an important peptide that is implicated in the control of motivated behaviours such as feeding and drinking. It acts on energy homeostasis through multiple pathways, including energy expenditure and locomotor activity [52]. In the SSC2_2 region, *PLAAT3* (phospholipase A/acyltransferase 3) is also an interesting candidate gene, as it is involved in the production, in the small intestine, of *N*-acylphosphatidylethanolamines (NAPE), which are implicated in the peripheral control of feed intake, causing a potent and persistent decrease in ingestion and gain in body mass when Oleylethanolamide is administrated [53]. More recently, Wang et al. [54] reported in pigs that lncPLAAT3-AS promoted adipocyte differentiation by acting as a sponge for miR-503-5p, thereby repressing the activity of this miRNA. Repression of miR-503-5p has also been shown to increase expression of PLAAT3, thereby promoting the lipogenic differentiation of porcine primary preadipocytes [54].

Heat stress also weakens gut integrity and compromises gut barrier function. This leaky gut syndrom leads to intestinal dysfunction, alterations in intestinal permeability, and pathophysiology responses such as inflammatory response [55]. Among the genes localized in the QTL regions identified in our study, an important subset is included in immunity response networks, such as cytokine signalling in immune system, adaptive immune system, and neutrophil degranulation. Most of these genes are located in the SSC7 QTL regions identified in our study, notably SSC7_3, which contains the swine leucocyte antigen (SLA) complex, and could affect our traits of interest in complex intertwined ways, given the diversity of signals that were detected in the ALL data set or the significance of GxE in this region. In addition, in the SSC1_1 region, the *LCN2* (Lipocalin 2) gene has been associated with antibacterial, anti-inflammatory, and protection against cell and tissue stress [56]. In 2022, Deis [57] reported that *LCN2* plays a role in thermogenesis and lipid metabolism, involving the COX2-PGE2 and mTOR signalling pathways. Inhibition of mTOR by rapamycin induces expression of the *LCN2* gene and protein secretion in the presence of insulin. In contrast, LCN2 deficiency diminishes the effect of mTORC1 inhibition of COX2, which affects thermogenesis genes, lipogenesis, and lipolysis.

Given the effect of heat stress in pigs on adipogenesis accompanied by increased triacylglycerol storage, some genes involved in lipid metabolism may also be good candidates. The *PTGES* (prostaglandin E2) gene, a lipid mediator implicated in inflammatory diseases and in regulation of lipolysis and adipocyte differentiation, and *SLC27A4* (solute carrier family 27, member 4), which is involved in intestinal uptake and absorption of fatty acids, are both located in the SSC1_1 region. In SSC2_1, a good candidate gene is the *PNPLA2* (patatin Like phospholipase domain containing 2) gene, which encodes an enzyme that catalyses the first step of the hydrolysis of triglycerides for β-oxidation-coupled energy production [58], as heat stress increases lipid accumulation by reducing the expression of adipolysis related genes [59].

### Consistency with previously reported QTL in pigs, chickens, and bovines

In pigs, few GWAS analyses have been performed to identify regions associated with response to Heat stress. In 2018, Kim et al. [11] performed GWAS for rectal temperature (TR) and skin temperature (TS) during heat stress in crossbred gilts from commercial lines (PIC maternal × Duroc terminal sire). Among the QTLs they identified, eight regions overlap or map close to (less than 5 Mb) the QTLs we identified either for rectal (SSC1_1, SSC3_1, SSC4_1, SSC8_2, SSC14_5 and SSC15_1) or skin temperature (SSC4_2 and SSC7_1) (see Additional file 6: Table S3). Of these 8 common regions, 7 were significant or suggestive in our analyses using the LW model, confirming some variability for these stress responses in European breeds and lines used in commercial crosses.

The economic and welfare implications of heat stress do not only affect the pig industry and, as a result, GWAS for heat stress traits have also been carried out in other livestock species (see Additional file 6: Table S3). In poultry, several GWAS have been conducted in commercial egg-laying lines to assess the impact of heat stress on production and find their genomic drivers. On GGA17 (at 7.0 Mb), in the orthologous region of our SSC1_1 QTL region, Wolc et al. [60] reported a QTL near the *DBH* (dopamine beta-hydroxylase) gene that explained more than 1% of the genetic variance in heat stress mortality. The SSC15_1 QTL was also detected in an orthologous position on GGA7 (28.1 Mb) for a QTL affecting heat stress tolerance in chicken [61]. Some of our QTL regions were also orthologous to QTLs for heat stress traits detected in cattle. On BTA16 (35 Mb), corresponding to our SSC10_1 region, a region affecting rectal temperature during heat stress has been reported in lactating Holstein cows [30]. In the region corresponding to the SSC14_1 QTL, Otto et al. [62] identified a significant signal on BTA17 (73 Mb) associated with rectal temperature variability in an experimental F2 *Bos taurus* × *Bos indicus* population.

In addition to GWAS, some genome scans for selection signatures have been performed to identify selection sweeps that reflect adaptation signals to environmental stress. These analyses were also mainly performed in poultry and cattle, and some of the regions reported in the literature for these species are orthologous to regions we detected. In poultry, selective sweeps on GGA10 (3.7 Mb) and GGA17 (10.2 Mb), orthologous to SSC7_6 and SSC1_1, respectively, were proposed to explain how evolutionary forces have shaped the patterns of genetic variation under harsh climate in Iranian native chickens [63]. In addition, SSC5_2 and SSC6_4 QTL are orthologous to regions on BTA5 (58 Mb) and BTA2 (126 Mb) identified in bovine as signatures of selection for environmental adaptation in zebu × taurine hybrids in an East African shorthorn zebu population [64]. The orthologous regions of SSC12_1 and SSC14_4, corresponding to BTA19 (42.25) and BTA26 (37.1 Mb), respectively, were also reported as containing selective sweeps using South African indigenous breeds [65] and indicine and taurine cattle breeds [66], respectively. In all these selective sweep regions, candidates genes associated with the family of heat shock proteins, which are involved in a universal and ancient mechanism of heat stress responses, were retained as good candidates in these studies: *HSPA5* (Heat Shock Protein Family A (Hsp70) Member 5) (GGA17: 10.19–10.2) and *DNAJA4* (DnaJ Heat Shock Protein Family (Hsp40) Member A4) (GGA10: 3.75–3.76) in poultry, *DNAJC8* (dnaJ (Hsp40) homolog, subfamily C, member 8) (BTA2: 125.14–125.17), *DNAJC14* (DnaJ Heat Shock Protein Family (Hsp40) Member C14) (BTA5: 57.43–57.44), *HSPB9* (Heat Shock Protein Family B (Small) Member 9) (BTA19: 42.25–42.26), *HSPA12A* (Heat Shock Protein Family A (Hsp70) Member 12A) (BTA26: 37.07–37.24). Thus, compared with results for production traits in different livestock species, for which numerous association analyses have been carried out, QTLs for resistance or response to heat stress appear to be more conserved between species. Exposure to extreme heat is a significant stress for most living species and similar physiological responses have already been reported, from yeast to human, suggesting a high degree of evolutionary conservation, which may explain this observation [67].

## Conclusions

The first striking outcome of our study is the large genetic variability that segregates in a European pig population for its response to heat stress, paving the way for substantial potential to select for response to heat. In comparison, limited genetic variability in the Creole breed was a limiting factor to identify alleles specific to the contrast between Large White and Creole responses. In addition, as QTL regions were identified for thermoregulation traits only, recording these phenotypes seem to be necessary to capture the individual variability of responses under different types of heat stress with sufficient power. Most of the detected QTL regions were common to responses to acute and chronic heat stress, but some were specific to one response or the other, so dedicated studies are needed to further decipher the underlying biological mechanisms that affect differences in resistance to each type of stress. Some of these QTLs could be prioritized for marker-assisted selection in breeding programs, e.g., using SSC7_3 for backfat thickness under heat stress. Finally, as a high level of consistency was found between the QTL regions we detected for thermoregulation responses and genomic regions previously reported for these traits and traces of selection in other species, translational studies are likely powerful approaches to further validate candidate genes proposed in our study. Such validations would also benefit from expression studies in similar population designs as used here, in order to evaluate to which extent genes and pathways are actually affected by heat stress.

## Supplementary Information

**Additional file 1: Figure S1. **Heat map example of linkage disequilibrium on chromosome 14. Linkage disequilibrium (r²)) computed from LD analyses (below the diagonal) and LA analyses (above the diagonal).

**Additional file 2: Table S1. **Genome-wide and chromosome-wide thresholds used for associations studies.

**Additional file 3: Figure S2. **Building QTL regions from significant SNP. The horizontal line is the chromosome, where positions of the SNP are indicated by vertical dashes. Significant SNP are large dashes. Three QTL regions are identified, the maxSNP being indicated with a star. The region highlighted in red corresponds to a QTL region with only one significant SNP. The region highlighted in grey corresponds to a QTL region with 2 significant SNP. The region highlighted in blue comprised 3 significant SNP.

**Additional file 4: Figure S3. **Proportion of variance explained by the QTL windows detected with all models and analyses. In orange, proportion of variance explained by the SNP with maximum − log10(P) for genome-wide significant QTL windows. In blue, proportion of variance explained by the SNP with maximum − log10(P) for suggestive QTL windows found in the significant QTL regions.

**Additional file 5: Table S2. **Suggestive QTL windows detected in the QTL regions

**Additional file 6: Table S3. **For each QTL region, list of previously published genes and regions associated with heat resistance. List of differentially expressed genes (DEG) from Huau & al (2024), porcine QTLs identified by Kim & al (2018) and orthologous avian and bovine regions with QTLs or selective sweeps associated with heat adaptation or resistance. For the DEGs, only the genes contained in the QTL regions were listed. For QTL regions and traces of selection, a tolerance of +/- 5 Mb on either side of the QTL region interval was retained in order to compare our results with the regions identified in independent studies performed in pig, poultry and cow.

## Data Availability

Research data underlying these results are publicly available at the data INRAE institutional collection under the 10.57745/TLKLRJ.
