# Supplementary figures and images for: Detection of genomic regions affecting thermotolerance traits in growing pigs during acute and chronic heat stress
Source: Genet Sel Evol. 2025 Sep 25;57:53. doi: 10.1186/s12711-025-00995-x (PMC12465502; doi:10.1186/s12711-025-00995-x)

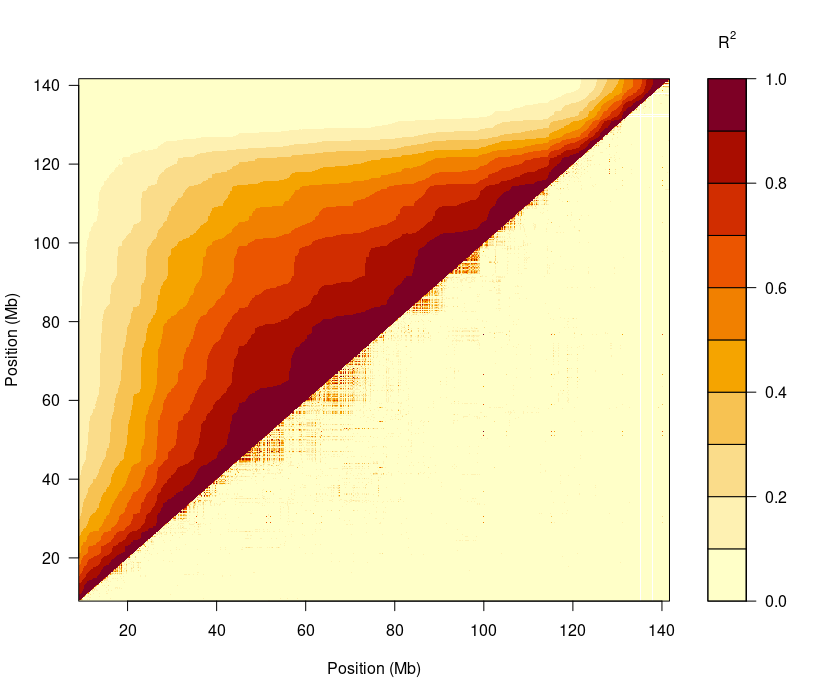

Supplement: Supplementary file 1 — Additional file 1: Figure S1. Heat map example of linkage disequilibrium on chromosome 14. Linkage disequilibrium (r²)) computed from LD analyses (below the diagonal) and LA analyses (above the diagonal). [file 12711_2025_995_MOESM1_ESM.bmp]

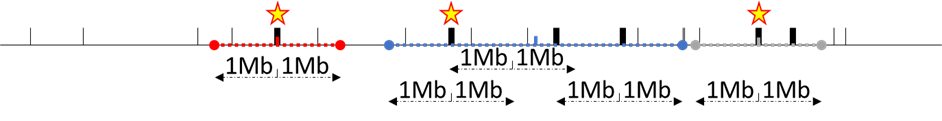

Supplement: Supplementary file 3 — Additional file 3: Figure S2. Building QTL regions from significant SNP. The horizontal line is the chromosome, where positions of the SNP are indicated by vertical dashes. Significant SNP are large dashes. Three QTL regions are identified, the maxSNP being indicated with a star. The region highlighted in red corresponds to a QTL region with only one significant SNP. The region highlighted in grey corresponds to a QTL region with 2 significant SNP. The region highlighted in blue comprised 3 significant SNP. [file 12711_2025_995_MOESM3_ESM.png]

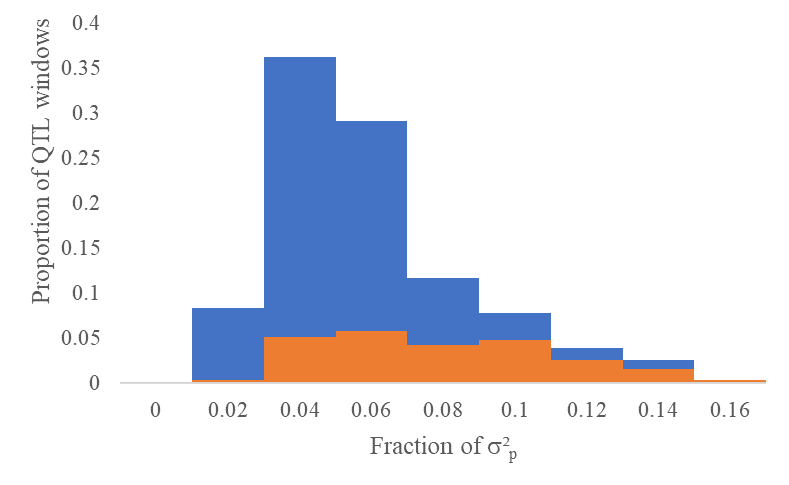

Supplement: Supplementary file 4 — Additional file 4: Figure S3. Proportion of variance explained by the QTL windows detected with all models and analyses. In orange, proportion of variance explained by the SNP with maximum − log10(P) for genome-wide significant QTL windows. In blue, proportion of variance explained by the SNP with maximum − log10(P) for suggestive QTL windows found in the significant QTL regions. [file 12711_2025_995_MOESM4_ESM.png]
